# Advances in Biomimetic Photoelectrocatalytic Reduction of Carbon Dioxide

**DOI:** 10.1002/advs.202203941

**Published:** 2022-08-25

**Authors:** Shaohan Xu, Qi Shen, Jingui Zheng, Zhiming Wang, Xun Pan, Nianjun Yang, Guohua Zhao

**Affiliations:** ^1^ School of Chemical Science and Engineering Key Laboratory of Spine and Spinal Cord Injury Repair and Regeneration, Ministry of Education, Tongji Hospital Tongji University Shanghai 200092 China; ^2^ Institute of Materials Engineering University of Siegen 57076 Siegen Germany; ^3^ Institute of New Energy, School of Chemistry and Chemical Engineering Shaoxing University 508 Huancheng West Road Shaoxing Zhejiang 312000 China

**Keywords:** CO_2_ conversion, electron transfer, photoelectrocatalysis, reaction mechanisms

## Abstract

Emerging photoelectrocatalysis (PEC) systems synergize the advantages of electrocatalysis (EC) and photocatalysis (PC) and are considered a green and efficient approach to CO_2_ conversion. However, improving the selectivity and conversion rate remains a major challenge. Strategies mimicking natural photosynthesis provide a prospective way to convert CO_2_ with high efficiency. Herein, several typical strategies are described for constructing biomimetic photoelectric functional interfaces; such interfaces include metal cocatalysts/semiconductors, small molecules/semiconductors, molecular catalysts/semiconductors, MOFs/semiconductors, and microorganisms/semiconductors. The biomimetic PEC interface must have enhanced CO_2_ adsorption capacity, preferentially activate CO_2_, and have an efficient conversion ability; with these properties, it can activate C=O bonds effectively and promote electron transfer and C—C coupling to convert CO_2_ to single‐carbon or multicarbon products. Interfacial electron transfer and proton coupling on the biomimetic PEC interface are also discussed to clarify the mechanism of CO_2_ reduction. Finally, the existing challenges and perspectives for biomimetic photoelectrocatalytic CO_2_ reduction are presented.

## Graphical abstract

Mimicking photosynthesis, photoelectrocatalysis (PEC) is a promising technique for CO_2_ conversion. Design strategies for building an efficient biomimetic PEC interface are summarized in this review, with a focus on the relationship between the interface functions and CO_2_ reduction reaction (CO_2_RR) performance. Moreover, the interfacial electron transfer and proton coupling are discussed to clarify the possible reaction pathways.

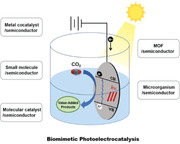

## 1 Introduction

As a notorious greenhouse gas and a potential carbon feedstock, the transformation of carbon dioxide (CO_2_) into high value‐added fuels or chemicals has been recognized as a promising way to mitigate energy shortages and problems caused by the greenhouse effect.^[^ ^1^ ^]^ Nevertheless, CO_2_ reduction is faced with several challenges. The solubility of CO_2_ in water under ambient temperature and pressure conditions is only 0.033 m,^[^ ^2^ ^]^ so it underperforms when competing with H_2_O molecules during the adsorption process. The low solubility and diffusion rate of CO_2_ in aqueous solution greatly restricts the efficiency of CO_2_ conversion. In addition, CO_2_ is a nonpolar linear molecule with two strong $\pi_{3}^{4}$ bonds; these bonds cause the energy of the C=O bond (750 kJ mol^–1^)^[^ ^3^ ^]^ to be significantly higher than that of the C—H bond and C—C bond. An immense amount of energy is required to break the C=O bonds owing to the molecular structure, which contributes to the relatively low conversion rate.^[^ ^4^ ^]^ In addition, the carbon atom in CO_2_ is in its highest oxidation state, which allows the CO_2_ reduction reaction (CO_2_RR) to go through various multielectron transfer processes, leading to poor product selectivity.^[^ ^5^ ^]^ Therefore, methods of enhancing the conversion rate and product selectivity of the CO_2_RR must be investigated.

**Figure 1:**
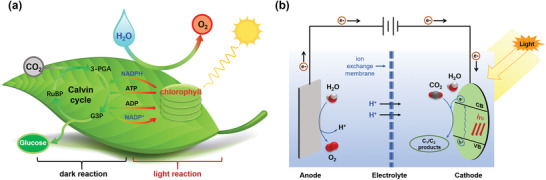
Schematics of a) natural photosynthesis and b) PEC CO_2_ reduction.

Natural photosynthesis in green plants is an efficient process in which atmospheric CO_2_ and H_2_O molecules are converted to glucose and oxygen through a moderate and efficient pathway via enzyme catalysis, sustaining the carbon‐oxygen cycle on Earth.^[^ ^6^ ^]^ It has been found that plant leaves have a perfect 3D hierarchical porous structure with high porosity, high connectivity, and a high specific surface area.^[^ ^7^ ^]^ These structures are not only conducive to the adsorption of sunlight but also favor the efficient migration of materials such as CO_2_ and H_2_O for photosynthesis and transpiration. Natural photosynthesis consists of two processes: light reactions and dark reactions (**Figure** **1**a). Atmospheric carbon dioxide and water first enter the interior of the leaves through the stomata on the surface of the leaves. In the light reaction, chlorophyll, the reaction center, harvests photons to generate electron‐hole pairs. The water is oxidized to oxygen by the photogenerated holes, providing electrons and protons for the fixation of CO_2_.^[^ ^8^ ^]^ Subsequently, in the dark reaction, CO_2_ is captured, activated and converted via a cyclic and directed electron transfer process, the Calvin cycle. The enzyme ribulose bisphosphate carboxylase/oxygenase (RuBisCO) enables CO_2_ to combine with ribulose 1,5‐bisphosphate (RuBP) to yield a six‐carbon compound, achieving the activation of CO_2_ molecules. This six‐carbon compound is extremely unstable and rapidly decomposes into two three‐carbon molecules, 3‐phosphoglycerate (3‐PGA). 3‐PGA is reduced by the NADPH produced from the light reaction, undergoes a series of complex proton‐coupled electron transfer (PCET) processes and is eventually converted to glucose.^[^ ^9^ ^]^

Inspired by natural photosynthesis, the photocatalytic reduction of CO_2_ mimicking the light reaction has been developed since the 1970s.^[^ ^10^ ^]^ Photocatalysis (PC) is considered a promising technique for CO_2_ reduction owing to the moderate reaction conditions and lack of extra energy input.^[^ ^11^ ^]^ However, PC still faces several problems, such as the uncontrollability of electron transfer, which results in the poor selectivity of the reduction products, the easy recombination of photogenerated electrons and holes,^[^ ^5^ ^]^ and the susceptibility of semiconductors to photocorrosion.^[^ ^12^ ^]^ Directed electron transfer, which can be achieved in the dark reaction in natural photosynthesis, is difficult to achieve through PC because the photocatalytic interface is a heterogeneous interface where the photogeneration of electrons is not cyclic; the photogenerated electrons are gradually consumed as the PC reaction proceeds. In contrast, electrocatalysis (EC) has the merits of strong controllability, since an electrochemical system is a closed and cyclic system where the electrons can move directionally upon application of an external voltage. In addition, protons can be generated at the anode and subsequently migrate to the cathode to participate in CO_2_ reduction. However, the EC process usually requires large amounts of electrical energy input owing to the high overpotentials of the CO_2_RR. Therefore, biomimetic PEC, which integrates PC and EC, can complement each other and achieve the thorough imitation of photosynthesis, providing a multiproton and multielectron cyclic coupling reaction mode (Figure 1b). The electrons can be produced from the light reaction, and directed electron transfer can be implemented by tuning the applied voltages, which mimics the dark reaction in photosynthesis. The applied potentials can effectively inhibit the recombination of photogenerated electrons and holes, enhancing the photocatalytic efficiency. Furthermore, solar energy can supply additional charge, lowering electricity consumption.^[^ ^13^ ^]^ The advantages and disadvantages of PC, EC, and PEC for CO_2_ reduction are listed in **Table** **1**.

**Table 1:** List of advantages and disadvantages of PC, EC and PEC for CO_2_ reduction

Type of catalytic methods	Advantages	Disadvantages
PC	Moderate reaction conditions	Easy recombination of photogenerated electrons and holes
	Energy saving	Susceptibility of semiconductors to photocorrosion
		Uncontrollability of electron transfer resulting in the poor product selectivity
EC	Strong controllability achieved by closed and cyclic electrochemical systems	High electrical energy consumption
	Feasible integration with other technologies	Decreased stability due to electrode passivation
	Easy to operate	
PEC	Suppression of the recombination of photogenerated electron–hole pairs	Relatively complex operation and equipment
	Lower electricity consumption	Further investigations required for the reaction mechanisms of PEC CO_2_ reduction
	High efficiency	

In addition to efficient photoelectron transfer and recycling, the reduction of CO_2_ in the dark reaction, which involves recyclable electron/proton transfer and CO_2_ adsorption, activation and conversion, is also notable. The preliminary step of the CO_2_ conversion process in natural photosynthesis is the adsorption of CO_2_ molecules. The leaves of green plants, which have a perfect 3D porous structure with high porosity, connectivity, and specific surface area, can efficiently transfer CO_2_ to the chloroplast stroma to complete photosynthesis through their stomata. The ultrahigh specific areas provide many active sites for CO_2_ adsorption. The porous structure is favorable for the mass transport of CO_2_ and H_2_O,^[^ ^14^ ^]^ and the light loss can be reduced owing to the reflection of incident light in porous structures.^[^ ^15^ ^]^ After the efficient transport and adsorption of CO_2_, the rapid activation of C=O bonds can be achieved by the RuBisCO enzyme. Following a series of complex electron transfer and proton coupling processes, the selective conversion of CO_2_ to glucose is accomplished. Moreover, during the fixation of CO_2_ in photosynthesis, the indispensable proton transferase NADPH is formed by the reduction and protonation of the electron acceptor NADP^+^ in the light reaction.^[^ ^16^ ^]^ The cyclic redox reaction between NADP^+^ and NADPH can supply recyclable protons and electrons for the CO_2_RR. Analogous to natural photosynthesis, a remarkable biomimetic PEC interface could adsorb, fix, and activate CO_2_ and recycle electrons and protons.

Unlike several excellent previous reviews focusing on the catalysts,^[^ ^5^, ^17^ ^]^ catalytic methods,^[^ ^18^ ^]^ and products^[^ ^19^ ^]^ of CO_2_ reduction, also those related to photoelectrocatalytic CO_2_ conversion which focuses on the functions and performance of a specific category of catalytic materials or the selection of semiconductors,^[^ ^20^ ^]^ strategies for selective CO_2_ conversion to different products,^[^ ^21^ ^]^ and reaction mechanisms,^[^ ^22^ ^]^ our review sheds light on strategies for constructing a biomimetic PEC interface to realize certain functions of photosynthesis, focusing on one or several factors to simulate natural processes. Most recently, a review focusing on structural design of artificial leaves has been published in which the concept of mimicking natural photosynthesis is introduced in terms of catalyst design resembling the structure and function of plant leaves.^[^ ^6^ ^]^ In this context, we redefine the PEC CO_2_ reduction system as a simulation of natural photosynthesis. The process by which a semiconductor is excited by illumination to produce photogenerated electron–hole pairs mimics the light reaction of photosynthesis. The directed electron transfer induced by the applied electric field mimics the dark reaction, which realizes the controllability of the electron transfer process by regulating the applied voltage. In this review, we propose several strategies for constructing an effective biomimetic PEC interface consisting of a metal cocatalyst/semiconductor, small molecule/semiconductor, molecular catalyst/semiconductor, MOF/semiconductor and microorganism/semiconductor. Enhanced CO_2_ adsorption capacity, effective CO_2_ activation and efficient conversion are essential for a remarkable biomimetic PEC interface so that the C=O bonds can be activated efficiently and electron transfer and proton coupling can occur. To better understand the underlying reaction mechanisms, the interfacial electron transfer process on different biomimetic PEC interfaces and possible reaction pathways involving proton coupling are elucidated. To this end, the existing challenges and future development of biomimetic PEC interfaces for CO_2_ reduction will be discussed and outlined.

## 2 Construction of Biomimetic Photoelectrocatalytic Interfaces

An excellent biomimetic PEC interface must exhibit prominent light absorption capacity, rapid photoelectric separation efficiency, high specific area for CO_2_ adsorption and abundant reactive sites for effective activation of CO_2_. Thus far, individual catalytic components do not yet meet all of the above requirements. The components have several limitations, such as inadequate contact between CO_2_ and the catalytic interface, poor carbon fixation performance, a substantial activation energy barrier, and unspecific product selectivity. Therefore, the effective assembly of catalysts with different functions is needed to complement the advantages of each. Building biomimetic PEC interfaces to achieve effective CO_2_ capture and fixation on one interface is a promising research direction. In this section, we aim to review different strategies for the construction of biomimetic PEC interfaces. These interfaces can be categorized as metal cocatalysts/semiconductors, small molecules/semiconductors, molecular catalysts/semiconductors, MOFs/semiconductors, and microorganisms/semiconductors (**Table** **2**).

**Table 2:** List of several biomimetic photoelectrocatalytic interfaces for CO_2_ reduction

Biomimetic functional interface								
Composition	Major products	Number of electrons transferred	Selectivity [%]	FE [%]	TON	Yield [µmol/g‐cat/h]	Quantum yield [%]	Refs.
Metal cocatalyst/semiconductor								
Pt/TiO_2_	CH_4_	8	N.A.	N.A.	N.A.	1361	2.41	[29]
Au‐Cu/P25	CH_4_	8	97	N.A.	N.A.	2200	N.A.	[38]
Cu@TiO_2_‐Au	HCOOH	2	98	82.6	N.A.	N.A.	N.A.	[39]
Au‐Cu/SrTiO_3_/TiO_2_	CO	2	N.A.	N.A.	N.A.	3770	N.A.	[40]
Au‐Pd/TiO_2_{101}	CH_4_	8	71	N.A.	N.A.	N.A.	N.A.	[41]
Rh LWs/TiO_2_	C_2_H_5_OH	12	N.A.	N.A.	N.A.	12.1	N.A.	[92a]
Au‐ZnTe/ZnO	CO	2	66.0	63.0	N.A.	N.A.	N.A.	[92b]
Pd/TiO_2_	CO, CH_4_	2, 8	10.1, 69.2	N.A.	N.A.	22.2, 38.1	N.A.	[92c]
Small molecule/semiconductor								
NH_3_/g‐C_3_N_4_	CH_4_, CH_3_OH	8, 6	N.A.	N.A.	N.A.	1.39, 1.87	N.A.	[47]
NH_2_‐C/Cu_2_O	HCOOH	2	92	N.A.	N.A.	138.65	N.A.	[48]
Amine‐functionalized graphene/CdS	CH_4_	8	N.A.	N.A.	N.A.	2.84	N.A.	[93a]
Amine/g‐C_3_N_4_	CH_4_, CH_3_OH	8, 6	N.A.	N.A.	N.A.	0.34, 0.28	N.A.	[93b]
Molecular catalyst/semiconductor								
[Ru‐dcbpy]/N‐Ta_2_O_5_	HCOOH	2	75	N.A.	89	N.A.	1.9	[56]
Ru(bpy)_2_dppz/Co_3_O_4_	HCOO^–^	2	99.95	86	N.A.	N.A.	N.A.	[57]
[Ru‐dpbpy]/N‐Ta_2_O_5_ anchored by PO_3_H_2_	HCOOH	2	N.A.	N.A.	118	N.A.	N.A.	[58]
CoTPP/g‐C_3_N_4_	HCOOH	2	100	N.A.	137	N.A.	N.A.	[64]
Coqpy@mesoporous graphitic C_3_N_4_	CO	2	98	N.A.	128	N.A.	0.25	[94b]
Re complex/CuInS_2_/NiO	CO	2	N.A.	32	11	N.A.	N.A.	[94c]
MOF/semiconductor								
ZIF8/Zn_2_GeO_4_	CH_3_OH	6	N.A.	N.A.	N.A.	0.22	N.A.	[71]
Cu_3_(BTC)_2_/TiO_2_	CH_4_	8	N.A.	N.A.	N.A.	2.64	N.A.	[72]
Co‐ZIF9/g‐C_3_N_4_	CO	2	86.3	N.A.	N.A.	495	0.9	[73]
Ni_3_(HITP)_2_/[Ru(bpy)_3_]^2+^	CO	2	97	N.A.	N.A.	34 500	N.A.	[95a]
Ni(II) MOF/g‐C_3_N_4_	CO, CH_4_	2, 8	N.A.	N.A.	N.A.	13.6	N.A.	[95b]
CTU/TiO_2_	CO	2	N.A.	N.A.	N.A.	31.32	N.A.	[95c]
UiO‐66/MoS_2_	CH_3_COOH	8	94	N.A.	N.A.	39.0	N.A.	[95d]
Microorganism/semiconductor								
*Sporomusa ovata*/Si nanowire	CH_3_COOH	8	N.A.	90	N.A.	N.A.	N.A.	[84]
*Methanosarcina barkeri*/*n* ^+^/*p*‐Si/NiMo	CH_4_	8	N.A.	82	N.A.	N.A.	N.A.	[86]
*Moorella thermoacetica*/AuNCs	CH_3_COOH	8	N.A.	N.A.	N.A.	34.76	2.86	[96a]

### 2.1 Metal Cocatalyst/Semiconductor Biomimetic Interfaces

The incorporation of cocatalysts cannot only favor the rapid separation of photogenerated electron–hole pairs but also accelerate sluggish reaction kinetics by lowering the overpotential of the CO_2_RR. The additional active sites provided by the metal cocatalyst can also facilitate the activation of the absorbed CO_2_ molecules. Cocatalysts with specific functions can be used to adjust the adsorption strength of certain intermediates, promoting the selectivity for the desired products.^[^ ^23^ ^]^ In addition, the side or back reactions can be suppressed by cocatalysts to further improve the product selectivity.^[^ ^24^ ^]^ Accordingly, cocatalyst deposition can be considered an effective way to enhance the activation and selective reduction of CO_2_.

**Figure 2:**
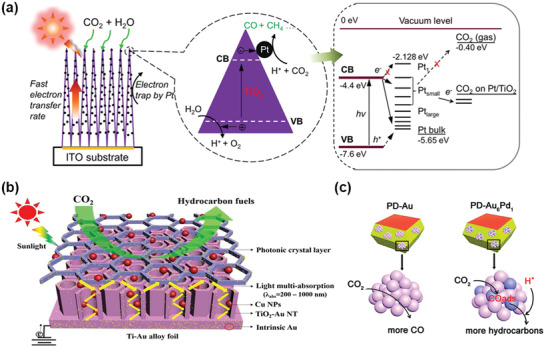
a) Schematic diagram of CO_2_ photoreduction mechanism by using Pt–TiO_2_ nanostructured films. Reproduced with permission.^[^ ^29^ ^]^ Copyright 2012, American Chemical Society. b) Schematic diagram for the synergy of the photonic crystal, Au NPs, and Cu NPs for CO_2_ reduction under light illumination. Reproduced with permission.^[^ ^39^ ^]^ Copyright 2017, Elsevier. c) A schematic illustration of the product distribution on PD‐Au and PD‐Au_6_Pd_1_. Reproduced with permission.^[^ ^41^ ^]^ Copyright 2019, Royal Society of Chemistry.

Metals such as Au, Ag, Pd, Cu, and Pt are among the most prevalent cocatalysts employed in PC and PEC CO_2_ reduction. The combination of a metal and a semiconductor can suppress the recombination of photogenerated electrons and holes. For example, deposition of 10 wt% Cu nanoparticles (NPs) on graphene oxide can suppress the recombination of charge carriers by enhancing charge separation at the metal/semiconductor interface and achieve the efficient activation of CO_2_ through single‐electron transfer from the d orbital of the metal to the π* orbital of C—O, increasing the yield of photocatalytic CO_2_ to methanol by nearly 60 times.^[^ ^25^ ^]^ The deposition of metal can also alter the light absorption range and enhance light absorption. For instance, TiO_2_ films modified by Au NPs can widen the light absorption range of TiO_2_ to the visible spectrum and increase the methane yield by 24 times.^[^ ^26^ ^]^ The broadening of TiO_2_ light absorption range might be attributed to the localized surface plasmon resonance (LSPR) effect of Au NPs, which is derived from the collective oscillations of the electrons in the vicinity of the plasmonic nanostructure induced by the incident light.^[^ ^27^ ^]^ Due to this effect, noble metal NPs can absorb visible light and inject the photoinduced electrons into the conduction band of semiconductor to produce the visible light responsive TiO_2_.^[^ ^28^ ^]^ In addition to the incorporation of versatile metals with semiconductors, the performance of metal/semiconductor hybrid catalytic interfaces is governed by the particle sizes of the metal cocatalysts. For example, an investigation of the influence of Pt NPs with different sizes on the photocatalytic performance of TiO_2_ showed that as the size of the Pt particles decreases, the work function of Pt shifts from the bottom of the VB of TiO_2_ to the CB and vacuum energy level, as shown in **Figure** **2**a.^[^ ^29^ ^]^ With the decrease in the size of Pt NPs (0.5–2 nm) and the work function, the maximum separation rate of electron‐hole pairs can be reached; in this study, the optimal photocatalytic performance was obtained. However, further decreases in the size of the Pt NPs can lead to a work function that is higher than the CB, which suppresses the transfer of electrons.

In addition to the size of metal NPs, the facet also plays an essential role in the performance of metal–semiconductor hybrid catalysts. Bai et al.^[^ ^30^ ^]^ investigated the facet selectivity of Pd cocatalysts in graphitic‐phase C_3_N_4_ (g‐C_3_N_4_) nanosheets and revealed that Pd{111} facets favored the reduction of CO_2_ more than Pd{100} facets. The theoretical results showed that the adsorption energy of CO_2_ in the presence of Pd{111} (*E*_a_  = 0.230 eV) is much higher than that with Pd{100} (*E*_a_  = 0.064 eV), demonstrating a better performance for CO_2_ adsorption on Pd{111}. In addition, the activation energy barriers of CO_2_ reduction can be lowered by the participation of Pd{111} facets within the range of 7.15 to 3.98 eV. Therefore, due to the merits of g‐C_3_N_4_, CO_2_ reduction can be favorable in the case of Pd{111} facets.

Bimetallic cocatalysts have attracted much attention because they can tune the surface adsorption energy of CO_2_ and improve the product selectivity because they combine the properties of different metals to achieve a synergistic effect. Different metals show different adsorption capacities for *CO_2_^•−^, *CO, *HCO, and other intermediates in the CO_2_ reduction process, leading to various CO_2_ reduction products. Typically, Au, Ag, and Zn weakly adsorb the CO_2_ activation intermediate *CO, and thus, the main reduction product is CO. The weak adsorption capacity of Sn, Hg, and Pb for *CO_2_^•−^ is favorable to the production of formic acid.^[^ ^31^ ^]^ With respect to Cu, the Cu—C bond strength when the intermediate *CO is adsorbed is moderate, favoring the conversion of CO_2_ to multielectron and multicarbon compounds.^[^ ^32^ ^]^ Therefore, the product selectivity of the CO_2_RR can be controlled by adjusting the ratio of metals with different functions in the design of metal alloy cocatalysts.

Bimetallic cocatalysts such as Ni–Ga,^[^ ^33^ ^]^ Cu–Ag,^[^ ^34^ ^]^ Cu–Au,^[^ ^35^ ^]^ Cu–Pd,^[^ ^36^ ^]^ and Mo–Bi^[^ ^37^ ^]^ exhibit a more remarkable CO_2_ electrocatalysis performance than single‐metal catalysts, as they integrate multiple functional components to achieve a synergistic effect. P25 decorated with Au–Cu NPs showed a better CO_2_ photocatalysis performance than Au/P25 and Cu/P25.^[^ ^38^ ^]^ The yield of methane on Au–Cu/P25 (2200 µmol g^−1^ h^−1^) was much higher than that on single‐metal decorated Au/P25 (210 µmol g^−1^ h^−1^) and Cu/P25 (280 µmol g^−1^ h^−1^). Au and Cu play different roles in the photocatalytic system; the LSPR effect of Au broadens the response range of materials to visible light, while Cu can achieve the specific adsorption of the reaction intermediate CO. Another example is the decoration of Cu NPs and Au NPs on TiO_2_ nanotube photonic crystals (NTPC), which leads to a 1019.3 µmol L^–1^ cm^–2^ formic acid yield in 8 h, almost 6.3 times higher than that on traditional TiO_2_ NTs (Figure 2b).^[^ ^39^ ^]^ During catalysis, the local surface plasmon resonance effect of the intrinsic Au NPs promotes the utilization of visible light at the catalytic interface. TiO_2_ NTPC can not only act as a light harvester but also provide a large specific area for the deposition of Cu NPs. Cu NPs also act as reactive sites and synergistically facilitate PEC CO_2_ reduction. The product selectivity can be further improved by tuning the composition of the bimetallic cocatalysts. For instance, Kang et al.^[^ ^40^ ^]^ analyzed the CO_2_ photocatalytic performance of different proportions of Au–Cu alloy‐modified SrTiO_3_/TiO_2_ nanotube arrays (STO/TiO_2_ NTAs). The main product of Au@STO/TiO_2_ NTAs for the reduction of CO_2_ was CO due to the weak adsorption capacity of Au for the intermediate CO. With the incorporation of Cu NPs, the selectivity of CO_2_RR to CO decreased, and the selectivity to hydrocarbons increased. Among them, Au_3_Cu@STO/TiO_2_ NTAs showed the best activity for reducing CO_2_ to hydrocarbons, and the performance was significantly better than that of the non‐alloy system (Au_3_+Cu_1_@STO/TiO_2_).

In addition to the incorporation of Au–Cu bimetallic cocatalysts, a Au–Pd alloy decorated on TiO_2_{101} facets was fabricated to enhance the conversion of CO_2_ to hydrocarbons (Figure 2c).^[^ ^41^ ^]^ The Au–Pd alloy can provide copious CO_2_ adsorption and activation sites with dispersed Pd atoms acting as hydrogenation centers. The cooperative combination of the Au–Pd alloy and the semiconductor TiO_2_ contributes to efficient CO_2_ reduction. Different elemental compositions of the Au–Pd alloy cocatalysts were explored for their photocatalytic CO_2_ reduction performance. PD–Au_6_Pd_1_ exhibited optimal CO_2_ conversion reactivity with a maximum hydrocarbon selectivity of 85%. To determine the reasons for the excellent performance of PD–Au_6_Pd_1_, further investigations were conducted. PD–Au_6_Pd_1_ exhibited a significantly higher CO_2_ adsorption ability, and an obvious LSPR absorption peak corresponding to Au was revealed by UV–vis diffuse reflectance spectroscopy. The presence of SPR and the corresponding plasmonic heat effect facilitated the activation of CO_2_ and lowered the energy barrier for the CO_2_RR.

Metal cocatalysts can effectively broaden the photoresponse range of semiconductors through localized surface plasmon resonance. The formation of a surface Schottky energy barrier can lead to the accumulation of photogenerated electrons on cocatalysts, facilitating the separation of photoexcited carriers.^[^ ^42^ ^]^ Moreover, the incorporation of metal cocatalysts can supply abundant active sites for both CO_2_ adsorption and activation, reducing the reaction energy barrier for CO_2_ reduction. For some cocatalysts with unique functions, the side reactions can be suppressed, and thus, the selectivity of the desired product can be enhanced. Nevertheless, factors such as the particle size and facet of metal NPs and composition and proportion of bimetallic cocatalysts should be taken into consideration when selecting and loading the cocatalysts, as these factors affect the performance of the catalyst and the product selectivity of the CO_2_RR.

### 2.2 Small Molecule/Semiconductor Biomimetic Interfaces

Despite the nonpolar linear structure of CO_2_ molecules, the surface charge distribution leads to positively charged C atoms and negatively charged O atoms, indicating the presence of both Lewis acid centers and Lewis base centers on the surface of the CO_2_ molecules.^[^ ^43^ ^]^ Hence, modification of the catalytic surface with acidic or basic molecules can improve the fixation and activation of CO_2_.

**Figure 3:**
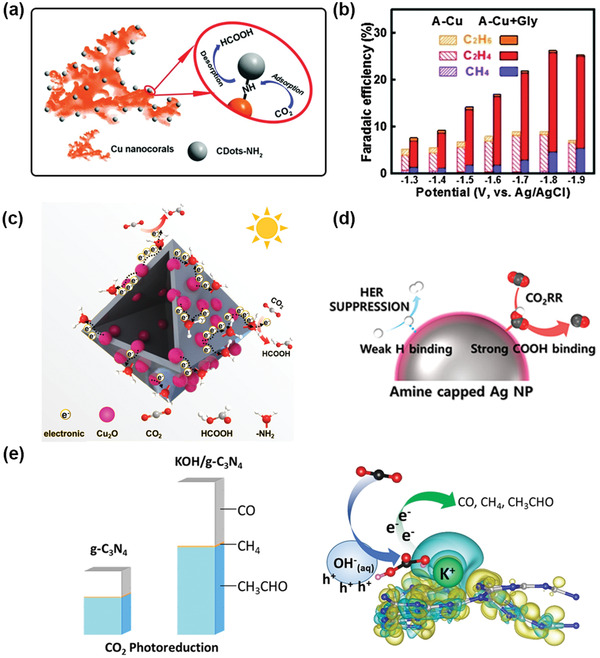
a) The proposed reaction mechanism of electrocatalytic CO_2_ reduction by Cu‐CDots nanocorals. Reproduced with permission.^[^ ^45^ ^]^ Copyright 2017, Royal Society of Chemistry. b) Electrochemical CO_2_ reduction on bare and glycine‐modified annealed Cu electrodes in the full potential range from −1.3 to −1.9 V. Reproduced with permission.^[^ ^46^ ^]^ Copyright 2016, Royal Society of Chemistry. c) Schematic illustration the process of charge migration and the proposed photocatalytic reduction of CO_2_ to HCOOH via the NH_2_‐C@Cu_2_O catalyst. Adapted with permission.^[^ ^48^ ^]^ Copyright 2021, Elsevier. d) Schematic of the HER and CO_2_RR process on Amine capped Ag NP. Reproduced with permission.^[^ ^49^ ^]^ Copyright 2017, American Chemical Society. e) The performance and effect of KOH decorated photoelectrode. Reproduced with permission.^[^ ^50^ ^]^ Copyright 2017, Elsevier.

Carbon dots (CDs), a carbon nanomaterial with various functional groups, can provide active sites for highly selective catalysis and linking groups to form connections with other materials.^[^ ^44^ ^]^ Cu–CD nanocorals synthesized by decorating carbon dots on Cu were reported to improve the adsorption of CO_2_ and reduce CO_2_ to formic acid at a low overpotential (0.13 V) with a 79% (‐0.7 V versus RHE) total Faradaic efficiency (FE) (**Figure** **3**a).^[^ ^45^ ^]^ The Fourier transform infrared (FT‐IR) spectrum and XPS results demonstrated that the carbon dot modification provided abundant functional groups, such as —OH, —NH—, and —C=O, on the surface of the Cu nanocorals. These functional groups enhanced the adsorption capacity of CO_2_ by 3.4 times (0.22 mmol g^–1^ at 25 °C) and improved the adsorption of H^+^. The concentrations of CO_2_ and H^+^ played a pivotal role in the PCET mechanism of CO_2_ reduction; thus, the increase in both concentrations on the catalyst surface directly led to the excellent CO_2_ catalytic performance of the Cu–CD nanocorals.

Amino acids are small molecules that can improve the stability of CO_2_ reduction intermediates on the catalytic interface. In one study, the reduction of CO_2_ was performed on Cu electrodes modified with different amino acids, which increased the hydrocarbon production selectivity on the Cu electrode.^[^ ^46^ ^]^ The results showed that all the tested amino acids enhanced the selectivity of CO_2_ to hydrocarbons, while different amino acids exhibited different abilities to increase the product selectivity. For example, modification with glycine maximized the selectivity of the CO_2_RR (Figure 3b). Theoretical investigations showed that modification with amino acids can facilitate the formation of hydrogen bonds between CO_2_ and —NH_3_^+^ in amino acid molecules, thereby stabilizing the two most important intermediates, COOH* and CHO*, in the conversion of CO_2_ to hydrocarbons.

An amine modification strategy can be employed to suppress the hydrogen evolution reaction (HER) and enhance the product selectivity of the CO_2_RR. One example focused on modifying and functionalizing the surface of g‐C_3_N_4_ with NH_3_ to obtain a hierarchical nanosheet that exhibited an enhanced light harvesting ability, abundant reactive sites, increased CO_2_ adsorption and improved separation of charge carriers.^[^ ^47^ ^]^ The N_2_ adsorption‐desorption isotherms showed that amine‐functionalized C_3_N_4_ nanosheets showed a much higher adsorption capacity than pristine C_3_N_4_ due to the presence of mesopores. The assembly of ultrathin C_3_N_4_ nanosheets led to the formation of an interconnected porous structure, which increased the specific area and facilitated mass transport. Amine decoration can enhance the CO_2_ adsorption ability via acid–base interactions between CO_2_ and the amine. Another study reported a threefold improvement in HCOOH yield on Cu_2_O functionalized with amino groups (Figure 3c).^[^ ^48^ ^]^ The enhancement can be attributed to both the strong chemisorption of CO_2_ by the interaction between the CO_2_ (acid) and —NH_2_ (base) and the suppression of the competing HER. In addition, as an electron acceptor, amino acids can accelerate charge transfer on the catalyst surface, inhibiting the recombination of photogenerated electrons and holes. The remarkable auxiliary function of the amine can also be verified on the EC interface. An example is the investigation of electrochemical CO_2_ reduction activity on Ag nanoparticle surfaces capped with oleylamine, oleic acid, and dodecanethiol.^[^ ^49^ ^]^ The results revealed that decoration with amines and thiols can both enhance the adsorption of CO_2_ to lead to the formation of a strong —COOH bond. However, modifications with thiols can also lead to a strong interaction with protons, which also strengthens the HER. Compared with thiol‐capped Ag NPs, the amine‐capped Ag surface destabilizes hydrogen binding, which suppresses the HER. Therefore, modifications with amines improved the CO production selectivity of the Ag nanoparticles (Figure 3d). The Faradaic efficiency of the CO_2_ to CO conversion on oleylamine‐capped Ag reached 94.2%, which was higher than that on oleic acid‐capped Ag (89.1%) and dodecanethiol‐capped Ag (65.5%).

Alkali metals are other small molecules that can be used to decorate semiconductors. They not only facilitate the activation of CO_2_ but also promote the separation of photogenerated electrons and holes to overcome one of the predominant shortcomings of semiconductors. For instance, KOH can be decorated on C_3_N_4_ to improve its performance for photocatalytic CO_2_ reduction (Figure 3e).^[^ ^50^ ^]^ The measurement of the CO_2_ reduction performance on C_3_N_4_ modified by KOH, KCl, K_2_CO_3,_ and KHCO_3_ indicated that K^+^ alone cannot promote the reaction. In a comparison of the product yield of the CO_2_RR on C_3_N_4_ modified with different concentrations of NaOH and KOH, the results showed that the yields of CO and CH_4_ on C_3_N_4_ decorated with KOH was 1.5 times that on C_3_N_4_ decorated with NaOH, indicating that the cations also influence the alkali metal‐decorated photocatalyst. The results of the photocurrent density experiments of g‐C_3_N_4_ in Na_2_SO_4_ solutions with different pH values illustrated that OH^–^ can act as a hole accepter to receive photogenerated holes and impede the recombination of photoexcited carriers. Theoretical calculation results show that although the adsorption energy of CO_2_ on K^+^ and Na^+^‐modified g‐C_3_N_4_ did not improve over that of the original g‐C_3_N_4_ (0.25 eV), the adsorption of H_2_CO_3_ on K/g‐C_3_N_4_ (1.13 eV) was 31% higher than that on Na/g‐C_3_N_4_ (0.86 eV). This result was ascribed to the position of K^+^ on the g‐C_3_N_4_; it tended to be located outside the pores, which was favorable for the adsorption of H_2_CO_3_. The stronger adsorption of H_2_CO_3_ may lead to the better photocatalytic performance of CN with KOH modification than with NaOH modification.

Small molecules employed for modifying the surface of semiconductors usually contain functional groups such as —OH and —NH—. These molecules all possess electron donors, which can provide adsorption sites for the electrophilic carbon atoms in CO_2_ and H^+^ from the electrolyte to activate and convert CO_2_.

### 2.3 Molecular Catalyst/Semiconductor Biomimetic Interfaces

In natural photosynthesis, chlorophyll, a magnesium porphyrin derivative, can harvest light and transform solar energy with porphyrin as the core photosensitive component.^[^ ^51^ ^]^ Mimicking the structure of chlorophyll, numerous researchers have dedicated their efforts to the development of molecular catalysts composed of various metal centers, especially transition metals, and ligands for CO_2_ reduction. Central multivalence transition metals in metal complexes can act as redox centers, which favors the implementation of multielectron transfer processes in the CO_2_RR.^[^ ^17b^ ^]^ Reaction intermediates can form via interactions between the metal centers and CO_2_ molecules, facilitating the reduction of CO_2_. Molecular catalysts can be roughly divided into four categories based on their ligands: 1) macrocyclic ligand metal catalysts, whose central metals are typically Co, Fe, Ni, and Cu;^[^ ^52^ ^]^ 2) bipyridine ligand metal complexes (Re, Ru, Mn, etc.);^[^ ^53^ ^]^ 3) phosphine ligand metal complexes (Rh, Pd);^[^ ^54^ ^]^ and 4) biomimetic metal complexes (Fe_4_S_4_ clusters).^[^ ^55^ ^]^ Molecular catalysts for the CO_2_RR typically possess the advantages of high product selectivity; thus, decorating them on the surface of semiconductors can further enhance the selectivity of the CO_2_RR. In addition, molecular catalysts can also act as photosensitizers to broaden the light response range of semiconductors.

**Figure 4:**
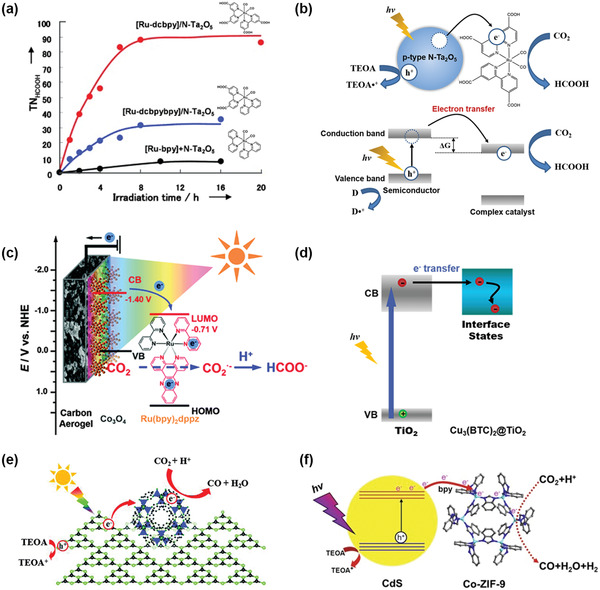
a) Turnover number for HCOOH formation from CO_2_ as a function of irradiation time on N‐Ta_2_O_5_ with different Ru complexes decorated in CO_2_‐saturated MeCN/TEOA (5:1) solution and b) energy diagram of hybrid photocatalysis under visible light with a semiconductor and a metal complex. Adapted with permission.^[^ ^56^ ^]^ Copyright 2010, Wiley‐VCH. c) Schematic plots of the CO_2_ adsorption‐enhanced Ru(bpy)_2_dppz‐Co_3_O_4_/CA interface together with its energy level diagram and the possible reaction pathways for CO_2_ conversion on this photocathode. Such a PEC interface is composed of CA as the CO_2_‐adsorption substrate, Ru(bpy)_2_dppz as the molecular catalyst, and Co_3_O_4_ as the photoelectrocatalyst. Reproduced with permission.^[^ ^57^ ^]^ Copyright 2016, Royal Society of Chemistry. d) Schematic illustration of the involved electron behavior. Reproduced with permission.^[^ ^72^ ^]^ Copyright 2014, Wiley‐VCH. e) The schematic diagrams of photocatalytic CO_2_ reduction on Co‐ZIF9/g‐C_3_N_4_. Reproduced with permission.^[^ ^73^ ^]^ Copyright 2014, Royal Society of Chemistry. f) The schematic diagrams of photocatalytic CO_2_ reduction on Co‐ZIF9/CdS. Reproduced with permission.^[^ ^74^ ^]^ Copyright 2015, Elsevier.

Among the aforementioned ligands, macrocyclic ligands (porphyrin, phthalocyanine, cyclam, etc.) and bipyridine ligands are the two predominant ligands investigated by researchers. One of the earliest examples is a series of Ru complexes ([Ru‐bpy], [Ru‐dcbpybpy] and [Ru‐dcbpy]) decorated on p‐type semiconductor N‐doped Ta_2_O_5_ to selectively reduce CO_2_ to formic acid under visible light in acetonitrile and triethanolamine solutions.^[^ ^56^ ^]^ **Figure** **4**a shows the TON_HCOOH_ of CO_2_ reduction on N‐Ta_2_O_5_ loaded with different Ru complexes in CO_2_‐saturated acetonitrile/TEOA solution over time. The experiment found that after decoration with the Ru complex, the TON of formic acid produced from the CO_2_RR catalyzed by N‐Ta_2_O_5_ was significantly enhanced. The [Ru‐dcbpy]/N‐Ta_2_O_5_ catalyst exhibited a selectivity of 75% for formic acid under 405 nm light, with a TON_HCOOH_ of 89 and a quantum efficiency of 1.9%. Photocatalytic CO_2_RR driven by the excitation of semiconductors by light to generate electrons that subsequently transfer from the conduction band to the ground state of the molecular catalysts was reported for the first time. The schematic diagram is shown in Figure 4b. The energy difference Δ*G* between the bottom of the semiconductor conduction band and the reduction potential of CO_2_ on the catalyst is an essential factor in photocatalytic CO_2_ reduction. Δ*G* can be optimized by changing the type of semiconductor, center atom, or ligand, thereby improving the photoactivity, selectivity, and stability of the catalyst. Adsorption capacity of the substrate is another important factor to be considered for the construction of efficient PEC interfaces. An example is a biomimetic interface for CO_2_ reaction integrating the light harvester Co_3_O_4_ and a ruthenium complex on the surface of a porous carbon aerogel, which has a high specific surface area (Figure 4c).^[^ ^57^ ^]^ Carbon aerogel and the enzyme‐mimicking ruthenium complex synergistically enhanced the concentration of surface‐adsorbed CO_2_. The Co_3_O_4_/ruthenium complex composite catalyst effectively absorbed light and produced electrons under light irradiation. The photoinduced electrons rapidly underwent directed transfer to CO_2_in the electric field. CO_2_ accepted the electrons and then underwent a two‐electron reduction process, which selectively converted it to formic acid. It was shown that this CO_2_ adsorption‐enhanced biomimetic catalytic system had several advantages, including low energy consumption (the CO_2_ reduction potential is only ‐0.45 V vs NHE), concentrated target product (formic acid selectivity>99%), and highly efficient conversion (Faradaic efficiency of 86%).

In addition, the connection between the semiconductor and the supporting molecules is also crucial for increasing the reaction rate. The immobilization of molecular catalysts on semiconductors in the aforementioned studies was realized by physical adsorption. The relatively feeble van der Waals interactions between molecular catalysts and semiconductors may lead to the leaching of catalysts, impairing the catalytic performance during long‐term PEC.^[^ ^17b^ ^]^ In addition, linkage via adsorption may reduce the efficiency of electron transfer between the metal complex and semiconductor. To achieve a strong linkage, phosphonate self‐assembly was employed to bind the semiconductor N‐Ta_2_O_5_ and [Ru‐dcbpy] to convert CO_2_ into formic acid under visible light.^[^ ^58^ ^]^ Different linking groups, including COOH and PO_3_H_2_, as well as different connection methods (anchoring with organic groups, physical adsorption and direct mixing), were investigated experimentally. The results showed that the main product on these four photocatalysts in the MeCN/TEOA (5:1, V/V) solution saturated with CO_2_ was HCOOH, and the two‐electron reduction product CO was also detected. Among them, the PO_3_H_2_‐linked catalyst exhibited the highest photocatalytic activity with a high TON_HCOOH_ of 118. This result indicated that the composite method and the chemical structure of the linking group can affect the photocatalytic activity of the catalyst.

Since then, an increasing number of molecular catalyst/semiconductor hybrid catalysts linked with different organic groups, such as cobalt porphyrin/conductive diamond linked by azide alkyne rings,^[^ ^59^ ^]^ carboxyl‐linked Re(CO)_3_‐Cl(dcbpy)/rutile TiO_2_{001},^[^ ^60^ ^]^ phosphate‐linked Ru(II) binuclear complexes/Ag/TaON,^[^ ^61^ ^]^ graphite‐conjugated *fac*‐Re(1,10‐phenanthroline)(CO)_3_Cl,^[^ ^62^ ^]^ and Re(bipy)/TiO_2_/Cu_2_O,^[^ ^63^ ^]^ have been investigated for photo/electrocatalytic CO_2_ reduction. This kind of valence‐bonded molecular catalyst/semiconductor system exhibits a rapid electron transfer channel, which can improve the quantum efficiency, TON and TOF of the CO_2_ reaction. To date, the most extensively investigated linking groups have been phosphoric acid groups, carboxyl groups and other oxygenic functional groups, which are beneficial for bonding with the metal on the semiconductor surface. However, the electron transfer efficiency of the systems containing these linking groups cannot outperform that of the systems with double bond and *π*–*π* stacking as the linking method. Liu et al.^[^ ^64^ ^]^ constructed a catalytic Co‐porphyrin/g‐C_3_N_4_ composite CO_2_ system by *π*–*π* stacking and realized the efficient adsorption and reduction of CO_2_ to the two‐electron product formic acid. The quasi‐3D structure of CoTPP provided pores that were slightly larger than CO_2_ molecules, which is suitable for the biomimetic adsorption of CO_2_. The electron cloud distribution indicates that the Co atoms in CoTPP can interact with O atoms in CO_2_ to activate CO_2_. The photoelectrochemical properties showed that CoTPP/g‐C_3_N_4_ exhibits excellent visible light absorption performance and PEC CO_2_ reduction capability. The conjugated *π*–*π* structure of CoTPP facilitates extending the visible light absorption range to approximately 700 nm, which is beneficial to the photoelectric reduction of CO_2_. Additionally, electrochemical studies have shown that it has good CO_2_ catalytic activity. The results revealed that it possessed a quasi‐3‐D structure similar to that of MOFs and a large specific surface area attributed to g‐C_3_N_4_, which favored the adsorption and reduction of CO_2_. In an aqueous electrolyte, at a low applied potential of ‐0.6 V, the amount of formic acid produced by photoelectric CO_2_ reduction for 8 hours can reach up to 154.4 µmol, and the TON can reach 137.

Molecular catalysts exhibit great advantages owing to the high controllability of their catalytic properties that can be gained by tailoring their molecular structures. The valence change of the central transition metal provides active sites for CO_2_ reduction. Combining molecular catalysts with semiconductor materials is a promising way to enhance the selectivity of the desired products, and the decoration of molecular catalysts can broaden the light response range of semiconductors as photosensitizers. Typically, electron transfer occurs inside hybrid catalysts; thus, determining how to achieve efficient, fast, and directed electron transfer between molecular catalysts and semiconductors is a compelling frontier for researchers. In addition, methods of tuning the molecular structures to achieve efficient and highly selective CO_2_ reduction are also worth investigating.

### 2.4 MOF/Semiconductor Biomimetic Interfaces

Metal organic frameworks (MOFs), a unique category of molecular catalysts, have attracted extensive attention due to their 3D porous structures. Generally, metal ions or clusters are used as connection nodes, and organic ligands support the construction of a 3D extension of space. After the emergence of zeolite and carbon nanotubes, MOFs have become an important new porous material with diverse structures and convenient design processes and are widely employed in catalysis, energy storage, and separation.^[^ ^65^ ^]^ Because their structure is similar to the hierarchical porous structure of plant leaves that enable efficient material migration and abundant adsorption sites for CO_2_ molecules, MOFs have an excellent adsorption capacity for CO_2_,^[^ ^65^, ^66^ ^]^ which can facilitate the further reduction of CO_2_. For example, a Cu‐based HKUST‐1 MOF supported on gas diffusion electrode (GDE) with high surface area was synthesized and employed in electrocatalytic CO_2_ reduction, achieving a 15.9% FE and a 17 h stability due to the preservation of its local structure.^[^ ^67^ ^]^ Furthermore, the comparison results with the other three different porous materials indicated that the unsaturated coordination sites exposed in the porous structure favor the conversion of CO_2_ to alcohols. In addition to the high surface area and robust structure, it is feasible to control and tune the local chemical environment of the active site in MOF to enhance its catalytic activity. A cationic functional group was tethered at the proximal end of the Fe‐porphyrin active site by postsynthetic modification to precisely tune the catalytic performance of a Fe‐porphyrin‐based MOF via electrostatic secondary–sphere interactions.^[^ ^68^ ^]^ The selectivity of electrochemical reduction of CO_2_ to CO was substantially enhanced to nearly 100% by immobilizing pendent positively charged groups. In situ Raman measurement revealed that the enhanced electrocatalytic performance was ascribed to the electrostatic stabilization of CO intermediates, boosting its desorption from the catalyst surface. Although MOFs are one category of promising materials for electrocatalytic CO_2_ reduction owing to the designable structures and dispersed metal active sites, their poor conductivity limits the enhancement of current density and performance. Yi et al. synthesized a conjugated 2D conductive phthalocyanine‐based MOF (NiPc‐NiO_4_) nanosheets as highly efficient electrocatalysts for CO_2_ conversion to CO.^[^ ^69^ ^]^ NiPc‐NiO_4_ exhibits good conductivity due to the high d‐*π* orbital overlap between the nickel node and the catechol, resulting in a nearly 100% CO selectivity and 34.5 mA cm^–2^ CO partial current density. MOFs can also be used as photocatalysts because of their photoresponse property. Wang et al. prepared three stable and isostructural MOFs (MOF‐Ni, MOF‐Co, and MOF‐Cu) for heterogeneous photocatalytic CO_2_ reduction.^[^ ^11b^ ^]^ Among them, MOF‐Ni displayed a remarkable selectivity of 97.7% for CO products. However, MOFs themselves have relatively low charge transfer efficiency when excited by light. Photocatalytic systems using single MOF‐based photocatalysts require the participation of a sacrificial electron donor.^[^ ^70^ ^]^

To enhance the limited charge transfer efficiency, thus far, several research groups have combined MOFs with semiconductors and utilized the light excitation ability of semiconductors and the CO_2_ adsorption and activation capacity of MOFs to improve the efficiency of CO_2_ reduction. For example, the amount of methanol generated by ZIF8/Zn_2_GeO_4_ composite photocatalytic CO_2_ reduction increased by 62% over that of pure Zn_2_GeO_4_ nanorods.^[^ ^71^ ^]^ Studies have shown that ZIF8 itself does not have photocatalytic activity for CO_2_, but it can effectively adsorb CO_2_ in aqueous solution and enhance the concentration of CO_2_ on the semiconductor surface. ZIF8 also broadens the photoresponse range of Zn_2_GeO_4_, thereby improving the photocatalytic CO_2_ reduction performance. Li et al.^[^ ^72^ ^]^ combined Cu_3_(BTC)_2_, which has CO_2_ adsorption capacity, with semiconductor TiO_2_ to prepare a Cu_3_(BTC)_2_@TiO_2_ core–shell structure that can efficiently reduce CO_2_ to methane via photocatalysis (Figure 4d). The conversion efficiency was 5 times as high as that catalyzed by pristine TiO_2_, and the ratio of methane to hydrogen increased from 22.7% to nearly 100%. Ultrafast transient absorption spectroscopy showed that after Cu_3_(BTC)_2_ was combined with semiconductor TiO_2_, the lifetime of photogenerated electron–hole pairs was effectively increased by more than 25 times. This result can be explained by the fact that after the combination, the photogenerated electrons can quickly migrate from TiO_2_ to the Cu_3_(BTC)_2_@TiO_2_ interface within 1 ps and participate in the reduction of CO_2_. ZIF9, another kind of ZIF that can catalyze CO_2_ reduction, was combined with a semiconductor to prepare two composite materials, Co‐ZIF9/g‐C_3_N_4_^[^ ^73^ ^]^ and Co‐ZIF9/CdS.^[^ ^74^ ^]^ Studies have corroborated that after semiconductor photocatalysts are combined with ZIF9, the ZIF9 assists in promoting the separation of photoinduced electrons and holes generated on the semiconductors. It can also promote the adsorption, capture, and fixation of CO_2_. Under visible light excitation, the semiconductor photocatalyst was excited to form photoinduced electron‐hole pairs. The photogenerated electrons then migrated to the CO_2_ adsorption sites on the MOF and participated in the conversion of CO_2_ to CO. A schematic diagram is shown in Figure 4e,f. By tuning the reaction conditions, such as solvent and temperature, the selectivity of CO_2_ reduction to CO in acetonitrile solution at 20 °C reached 91.2%.

Due to their abundance in the earth's crust, low cost, and unique properties for stabilizing reaction intermediates and promoting C—C coupling, Cu‐based catalysts in either Cu^2+^ or Cu^+^ form have been widely investigated for CO_2_ reduction. For instance, a multi‐shelled CuO microboxes electrocatalyst was constructed and achieved a FE of 51.3% owing to the approachability of catalytic active sites and enhanced adsorption of CO intermediates.^[^ ^75^ ^]^ Compared with Cu and CuO, Cu_2_O NPs display a good performance for generating C2+ products probably because the existence of low‐coordinated Cu^+^ species on the surface can facilitate the C—C coupling.^[^ ^76^ ^]^ For example, a 59% FE for the electroreduction of CO_2_ to ethylene was achieved on Cu_2_O NPs enclosed with both {111} and {100} facets.^[^ ^76b^ ^]^ The joint interface between two facets possesses strong adsorption capability of CO intermediates to promote C—C coupling and weaker adsorption ability of the generated C_2_H_4_ to facilitate its desorption from the interface. As the p‐type semiconductors, CuO and Cu_2_O possess relatively narrow band gaps and exhibit outstanding photocatalytic activity by visible light irradiation.^[^ ^77^ ^]^ Reduced graphene oxide–CuO nanocomposites were prepared for photocatalytic CO_2_ reduction to methanol with a yield of 1228 µmol g^–1^.^[^ ^78^ ^]^ In another study, Cu_2_O nanocrystals were employed in photocatalytic reduction of CO_2_, producing methanol as the sole product with a 72% internal quantum yield and 10% solar‐to‐fuel efficiency.^[^ ^79^ ^]^ The (110) facets of the prepared Cu_2_O nanocrystals are probably Cu terminated, which accounts for their high photocatalytic activity. However, bare copper oxides are susceptible to photocorrosion during photocatalysis or photoelectrocatalysis, resulting in reduced catalytic activity. The porous framework of MOFs can protect unstable semiconductors from photocorrosion. For instance, a core–shell structure with Cu_3_(BTC)_2_ MOFs decorated on Cu_2_O nanowires was constructed to improve the photocatalytic activity for CO_2_ reduction to CH_4_.^[^ ^80^ ^]^ Encapsulation in MOFs can protect unstable Cu_2_O from photocorrosion, and the semiconductor can enhance the light absorption capacity of the MOFs. With the combination of Cu‐based MOFs, the yield of CH_4_ can reach approximately 0.73 µmol for 8 h, which is 1.9 times higher than that of pristine Cu_2_O. The reusability of the catalysts was investigated, and it was found that the incorporation of MOFs led to a higher catalytic stability and prolonged durability. In addition, the porous structure attributed to the MOFs increased the surface‐to‐volume ratio, which enhanced the CO_2_ adsorption capacity. The EIS results indicated that the hybrid catalyst possessed a lower charge transfer resistance than bare Cu_2_O, demonstrating a faster charge transfer at the interface.

MOFs materials have a unique spatial 3D porous structure analogous to that of plant leaves, and this structure provides sufficient adsorption sites for molecules and high connectivity. By changing the central metal atom, tuning and modifying ligands and applying other manipulation methods, supramolecular interactions such as hydrogen bonds and *π*–*π* bonds can be endowed to the pores, thereby enhancing the adsorption and activation of CO_2_. To date, the combination of MOFs with semiconductors are mostly realized by direct mixing and physical adsorption, which leads to low charge carriers transfer efficiency between semiconductors and catalysts. Therefore, like the molecular catalyst/semiconductor catalytic system, the efficient and swift electron transfer between MOFs and semiconductors is also a frontier for researchers.

### 2.5 Microorganism/Semiconductor Biomimetic Interfaces

Since biological systems provide a relatively complete environment for utilizing solar energy in a more complex and effective way, microbial catalytic reduction of CO_2_ has unique advantages. Biological enzymes such as carbon monoxide dehydrogenase (CODHs), formate dehydrogenase (FDH), alcohol dehydrogenase (ADH), and formaldehyde dehydrogenase (FADH) can selectively convert CO_2_ to specific products at room temperature and atmospheric pressure. However, these reaction processes usually require expensive NADH as an auxiliary material.^[^ ^81^ ^]^ In addition, the shortcomings of the biophotosynthetic system are also obvious, since the primary goal of biophotosynthesis for plants is survival instead of maximizing the conversion of solar energy. The photosynthesis efficiency of most plants is only 0.1%, while the highest efficiency is no more than 6%.^[^ ^82^ ^]^

Microbial electrosynthesis (MES) is a new bioelectrochemical technique developed over the past decade. Microbial electrosynthesis of CO_2_ refers to a process driven by external electrical energy in which electrochemically active carbon‐fixing microorganisms take in CO_2_ as their only carbon source to convert it into value‐added chemicals and fuels. Currently, the most common microorganisms used for CO_2_ reduction via MES include methanogens and acetogens.^[^ ^83^ ^]^ These microorganisms can all convert CO_2_ with specificity at a relatively low cathode potential that is usually lower than ‐1.0 V versus SHE. Therefore, the combination of semiconductor nanocatalysts that possess excellent spectral absorption and photoelectric conversion capabilities with microorganisms can enhance the advantages of both to carry out the biomimetic PEC conversion of CO_2_.

**Figure 5:**
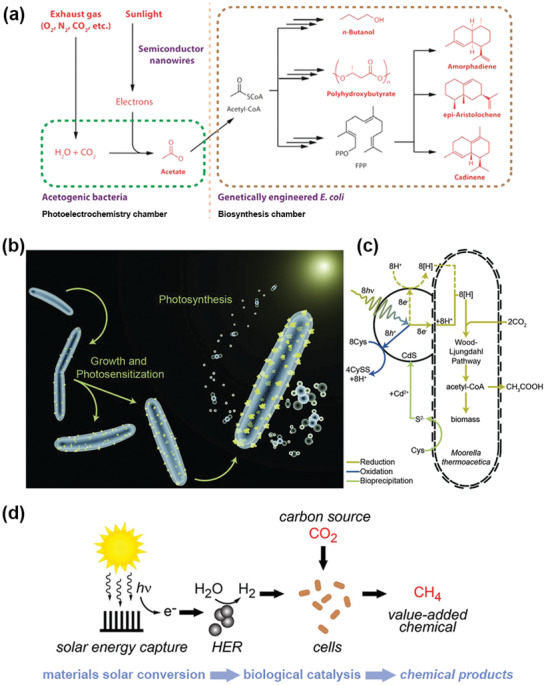
a) Schematic of a hybrid semiconductor nanowire‐bacteria system which can convert exhaust gas into liquid fuels, biopolymers, and pharmaceutical precursors. Reproduced with permission.^[^ ^84^ ^]^ Copyright 2015, American Chemical Society. b) Depiction of the *M. thermoacetica*–CdS hybrid system, proceeding from the growth of the cells and bioprecipitation (loading) of the CdS nanoparticles (shown in yellow) through photosynthetic conversion of CO_2_ (center right) to acetic acid (right), and c) pathway diagram for the *M. thermoacetica*–CdS system. Two possible routes to generate reducing equivalents, [H], exist: generation outside the cell (dashed line) or generation by direct electron transport to the cell (solid line). Reproduced with permission.^[^ ^85^ ^]^ Copyright 2016, American Association for the Advancement of Science. d) General scheme depicting a hybrid bioinorganic approach to solar‐to‐chemical conversion. Sustainable energy inputs in the form of electrical potential or light can be used to generate hydrogen from water using inorganic HER catalysts; biological hydrogen‐driven CO_2_ fixation can subsequently generate value‐added products such as methane. This materials biology interface can be generalized to other chemical intermediates and end products by mixing and matching different compatible inorganic and biological components. Adapted with permission.^[^ ^86^ ^]^ Copyright 2015, National Academy of Sciences.

There are currently two microbial/semiconductor catalysis systems for CO_2_ reduction that have different sources of electrons. In one system, microorganisms directly obtain electrons from the surface of semiconductors and then react with protons and CO_2_ inside the microorganisms. A hybrid semiconductor nanowire‐bacteria system with anaerobic bacterium and *Sporomusa ovata* fixed on Si nanowire arrays was constructed (**Figure** **5**a). This system utilized Si nanowires to capture light, providing energy for microorganisms and reducing CO_2_ to acetic acid at a relatively low overpotential (less than 200 mV) with a Faradaic efficiency as high as 90%; good stability was maintained even after 200 h of operation.^[^ ^84^ ^]^ This method can also be used to endow nonphotosynthetic microorganisms with photosynthetic abilities. After the combination of *Moorella thermoacetica* and the photocatalyst CdS, the photoelectrons generated on CdS can be used to convert CO_2_ to acetic acid with a high quantum efficiency of up to 85% ± 12% (Figure 5b,c).^[^ ^85^ ^]^ In the other system, microorganisms take the H_2_ generated from photocatalysis on the semiconductor and use it as an electron carrier to reduce CO_2_ inside the microorganisms. A hydrogen evolution electrocatalyst was used as the cathode to split water to produce hydrogen, and the highly biocompatible *Methanosarcina barkeri* was chosen as a biocatalyst for CO_2_ fixation so that H_2_ and CO_2_ could react in the microorganisms to produce CH_4_ (Figure 5d). The system achieved a total Faradaic efficiency of 86% over 7 d. After the anode and cathode were replaced with an InP photocathode and a TiO_2_ photoanode that possessed photocatalytic activity, the reaction could be driven under light without external input of electrical energy, and a FE of 82 ± 10% was achieved on the n^+^/p‐Si/NiMO photocathode with a low overpotential of 175 mV.^[^ ^86^ ^]^ Similarly, in the CoP_i_|Co‐P|*Ralstonia eutropha* hybrid system, *R. eutropha* bacteria can also utilize H_2_ generated by water splitting to convert CO_2_ into alcoholic liquid fuels and biomass. The energy efficiency of CO_2_ reduction has reached 10%, which is higher than that of the natural photosynthesis system.^[^ ^87^ ^]^ The main difference between these two types of transformation systems is the method of electron transfer. Microbial systems that directly accept electrons from electrodes cannot be applied with excessively high current density during operation, since high current density can lead to the rapid generation of hydrogen and the shedding of microbial membranes. These phenomena inhibit electron transfer and lead to a slower reaction rate. In contrast, indirect electron transfer is limited by the solubility of H_2_ in water, resulting in a low mass transfer efficiency.

Microbial/semiconductor biomimetic PEC can efficiently and selectively convert CO_2_ over a long operating lifetime. However, due to the complexity of microbial systems, it is necessary to consider the biocompatibility of the corresponding semiconductor materials and the interactions between the microorganisms and semiconductors. Moreover, it is also essential to understand the mechanisms of extracellular electron transfer; such an understanding can be achieved by integrating knowledge from multidisciplinary fields such as electrochemistry, materials science, engineering, microbiology, metabolic engineering, and synthetic biology. Therefore, the directed conversion of CO_2_ into fuels and value‐added chemicals can be improved.

Among these five semiconductor‐based biomimetic catalytic interfaces, metal cocatalysts have been widely studied. They can widen the photoresponse range of semiconductors effectively and promote the rapid separation of photogenerated carriers, but more importantly, their nanoparticle morphology and size can be controlled and their crystal facets can be adjusted, enabling the efficient activation of CO_2_. The activation configuration of CO_2_ is limited by the metal species, resulting in different selectivities of CO_2_ reduction products with different metal catalysts. However, compared with the other four types of biomimetic catalytic interfaces, the amount of CO_2_ adsorption at the metal interface is insufficient. The advantage of small molecule catalysts is that they can be designed with —OH, —NH—, and other functional groups to introduce acid–base active sites, thereby enhancing the adsorption and activation of CO_2_ at the interface. Molecular catalysts adsorb and activate CO_2_ through the weak coordination bond between the central metal and CO_2_ and its reduction intermediates. Such bonding clarifies the CO_2_ reduction mechanism on molecular catalysts, but the products are often limited to C1 products such as CO and HCOOH. By changing the central metal atom and regulating and modifying the ligands, acid–base active sites and supramolecular forces such as hydrogen bonds and *π*–*π* bonds are imparted to the porous 3D structure of MOF materials. Therefore, the adsorption and activation of CO_2_ in the pores of MOFs are enhanced. The MOF/semiconductor biomimetic interface combines the advantages of the above three types of biomimetic catalytic interfaces. It is designable in terms of light absorption and CO_2_ capture, adsorption, and activation. It is currently the most promising interface to fully simulate natural photosynthesis. The biomimetic microbial semiconductor interface uses microorganisms to metabolize CO_2_ to convert CO_2_. Therefore, this type of biomimetic interface clearly enables the formation of CO_2_ reduction products and it is expected to yield products with more carbon atoms. However, its shortcomings are also obvious; the CO_2_ reduction products and reduction efficiency are determined by the metabolism of microorganisms, so the selection and cultivation of microorganisms and their harsh growth environments are limiting factors of the system.

Although biomimetic photoelectrocatalytic reduction of CO_2_ is attracting extensive attention, the stability of photocathode has always been an important factor restricting the activity of catalytic CO_2_. Under illumination condition, electrons and holes can be formed on the semiconductor catalyst; however, these electrons and holes may induce photocorrosion due to the reduction or oxidation of the catalyst itself. Although the addition of voltage can alleviate photocorrosion, it is still a major problem that restricts the stability of catalysts. Researchers have made great efforts to find ways to enhance the stability of photocathodes. The ways to enhance the stability of the photocathode are summarized as follows. First, metal doping is an effective measure to mitigate photocorrosion. Metal doping on semiconductors enables the transfer of photogenerated electrons to metals and prevents the reduction of semiconductor catalysts. Liu et al. showed that photoelectrocatalytic CO_2_ conversion to ethylene can be achieved by electrodeposition of Ag onto Cu_2_O with ≈60% Faradaic efficiency for hours, whereas bare Cu_2_O degrades within minutes.^[^ ^88^ ^]^ Second, by constructing heterojunctions with other semiconductor materials, photocorrosion can be alleviated and the stability of catalysts can be enhanced. The Cu_2_O has strong photocorrosivity, which significantly affects the stability of catalysts. Zhang et al. loaded SnO*_x_* onto Cu_2_O nanowires (NWs) as photocathode for PEC reduction of CO_2_ to CO, which maintained long‐term stability within 12 h.^[^ ^89^ ^]^ Third, the unstable catalyst material can be encapsulated within the stable material to improve the stability of the catalyst. Wu et al. encapsulated Cu_2_O nanowires in MOFs of Cu_3_(BTC)_2_ (BTC = 1,3,5‐benzene tricarboxylate) for CO_2_ reduction.^[^ ^80^ ^]^ MOFs can not only suppress Cu_2_O corrosion induced by water vapor, but also promote charge separation and CO_2_ adsorption, resulting in 1.9 times increase in the activity and stability of the nanocomposite for selective photocatalytic CO_2_ reduction to methane. Fourth, the preparation method of the photoelectrode is also a factor affecting its stability. Currently, most of the catalyst materials are dispersed in Nafion and coated on the support electrodes (e.g., carbon paper, glassy carbon, fluorine‐doped tin oxide), whereas this loading method may lead to poor catalyst stability due to uneven contact or weak binding between catalysts and substrates. The connection methods such as *π*–*π* stacking^[^ ^64^ ^]^ and in situ growth^[^ ^90^ ^]^ can enhance the interaction between catalysts and substrates, thus improving photoelectrode stability and boosting charge transfer. More and more approaches are being explored to improve the stability of the photoelectrode for CO_2_ reduction. Solving the problem of photoelectrode stability will lay the foundation for the rapid development of biomimetic photoelectrocatalysis of CO_2_.

## 3 Electron Transfer and Proton Coupling on the Biomimetic Photoelectrocatalytic Interface

In natural photosynthesis, water is oxidized by photogenerated holes to produce oxygen and provide protons for the fixation of CO_2_ in the subsequent dark reaction.^[^ ^8^ ^]^ CO_2_ is then reduced via the Calvin cycle, which is a directed electron transfer process. Similar to natural photosynthesis, water can be oxidized at the anode of the biomimetic PEC interface, producing oxygen and protons.^[^ ^23^ ^]^ Subsequently, CO_2_ is reduced on the cathode via a directed electron transfer and proton coupling process to generate various products. This process is accompanied by an intricate mechanism involving multielectron transfer and proton coupling processes, resulting in poor product selectivity. Considering the complexity of the reaction on the biomimetic PEC interface, clarifying the electron transfer process between catalysts and semiconductors and elucidating the potential pathways of the CO_2_RR are of great importance. In this section, we illustrate the interfacial electron transfer process on different biomimetic PEC interfaces and summarize several possible reaction pathways for the reduction of CO_2_ to different products. Moreover, proton coupling on the PEC interfaces is discussed according to different proton sources.

### 3.1 Electron Transfer and Product Regulation

As mimics of natural photosynthesis on the hybrid biomimetic PEC interface, modified materials, including metals, small molecules, molecular catalysts, MOFs and microorganisms, typically perform as active sites for CO_2_ adsorption, activation and reduction, while semiconductors absorb light and supply active photogenerated electrons to facilitate the CO_2_RR. CO_2_ is reduced by a directed electron transfer process on the biomimetic PEC interface. In contrast to PC and EC, the electrons on the PEC interface originate from two pathways: some electrons are photogenerated, while others are supplied by applied voltages. Under light irradiation at a specific wavelength, the semiconductor photocathode is excited and produces photogenerated electrons in the conduction band and holes in the valence band.^[^ ^91^ ^]^ The applied electric field can facilitate the separation of charge carriers and drive the migration of photogenerated electrons to the surface of the cathode. Then, CO_2_ is reduced to fuels on the cathode by the photogenerated electrons and the electrons provided by the applied voltage.

**Figure 6:**
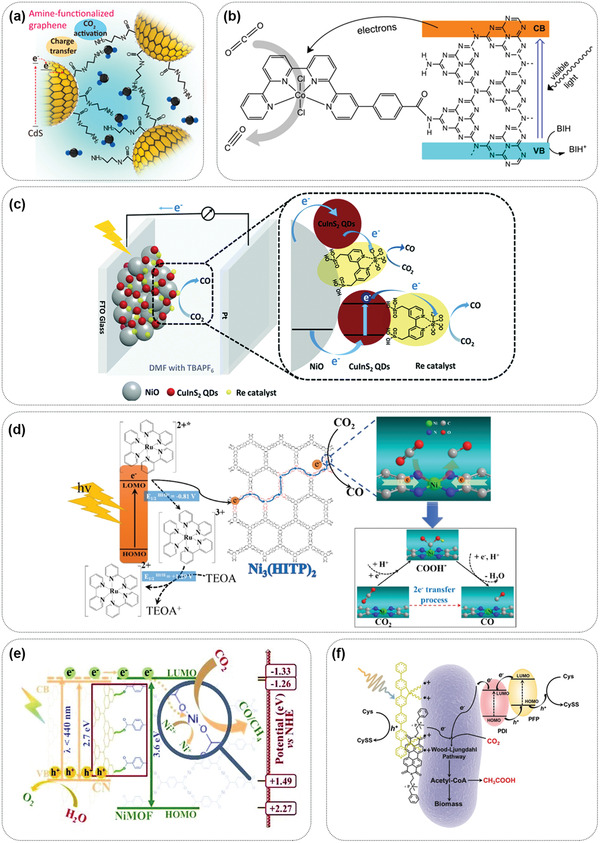
a) Graphical illustration of the proposed mechanism of CO_2_ photoreduction on the AG/CdS. Reproduced with permission.^[^ ^93a^ ^]^ Copyright 2017, American Chemical Society. b) Illustration of the visible‐light‐driven CO_2_ to CO reduction process on Coqpy@mesoporous graphitic C_3_N_4_. Reproduced with permission.^[^ ^94b^ ^]^ Copyright 2020, American Chemical Society. c) Configuration of a PEC cell for CO_2_ reduction based on the NiO photocathode cografted with the CuInS_2_ QDs and the Re catalyst, and illustration of electron injection and hole transfer in the photocathode. Reproduced with permission.^[^ ^94c^ ^]^ Copyright 2019, Royal Society of Chemistry. d) Proposed Mechanism of photocatalytic CO_2_ reduction to CO with Ni_3_(HITP)_2_ under visible‐light irradiation. Reproduced with permission.^[^ ^95a^ ^]^ Copyright 2018, Elsevier. e) A schematic diagram of the photogenerated charge transfer process and the induced photochemical reaction in the resultant NiMOF/functionalized CN nanocomposite. Reproduced with permission.^[^ ^95b^ ^]^ Copyright 2020, Royal Society of Chemistry. f) Diagram of the photoexcited electron generated from PDI/PFP under illumination transferred by the membrane protein and finally passed on to the Wood–Ljungdahl pathway for CO_2_ reduction. Reproduced with permission.^[^ ^96b^ ^]^ Copyright 2020, Wiley‐VCH.

Interfacial electron transfer on different biomimetic PEC interfaces can be achieved by the potential difference between the catalyst and semiconductor, where the transfer driving force consists of the Schottky barrier, the difference between the conduction band minimum and the lowest unoccupied molecular orbital (LUMO) potential. At the metal cocatalyst/semiconductor biomimetic PEC interface, the Schottky junction between the metal particles and the semiconductor can promote the separation of charge carriers and cause the photogenerated electrons to accumulate on the metal cocatalysts. The electrons can then be transferred from the semiconductor to the cocatalyst under photoinduction, which is followed by the reduction of CO_2_.^[^ ^92^ ^]^ At the small molecule/semiconductor biomimetic interface, small molecules act as electron donors and provide adsorption sites for the electrophilic C atoms of CO_2_, activating it. The supplementation of photogenerated electrons from the semiconductor can further improve this effect and reduce CO_2_ (**Figure** **6**a).^[^ ^93^ ^]^ A molecular catalyst can be designed to precisely control the functionality, and it can act as the reduction center when a semiconductor is used as the light absorber, forming an efficient hybrid catalytic system. At the molecular catalyst/semiconductor biomimetic PEC interface, the semiconductor absorbs light to generate photoinduced electrons, which are transferred to the molecular catalyst along with the electrons emanating from the external circuit due to the match between the minimum position of the semiconductor conduction band and the potential of the lowest unoccupied molecular orbital (LUMO) of the molecular catalyst (Figure 6b,c).^[^ ^94^ ^]^ MOFs are a unique category of molecular catalysts. The interfacial electron transfer at the MOF/semiconductor biomimetic PEC interface exhibits a trend similar to that of aforementioned molecular catalyst/semiconductor interface, implying that electron transfer from the semiconductor to the MOFs occurs (Figure 6d,e).^[^ ^95^ ^]^ At the microorganism/semiconductor PEC interface, electron transfer proceeds from the (photo)electrode to the biocatalyst. The semiconductor and the external bias provide electrons for the biosynthesis process, while the microorganisms exhibit superior catalytic performance for CO_2_ reduction (Figure 6f).^[^ ^96^ ^]^

After interfacial electron transfer from the semiconductor to the catalytic surface, the adsorbed CO_2_ is reduced via a series of electron transfer processes. Since the carbon atom in CO_2_ is in its highest valence state, a wide range of reduction products can form. The CO_2_RR may go through different reaction pathways to reach the same product by generating different intermediates. The reduction product depends on the number of transferred electrons, and the potential reaction pathway is related to the catalytic interface. After the formation of a bent CO_2_•^−^ anion radical via a one‐electron reduction of CO_2_, the intermediate *COOH is generated via the protonation of the oxygen atom. Following one electron transfer process with subsequent desorption from the catalytic surface, CO is released.^[^ ^97^ ^]^ Alternatively, CO_2_•^−^ may also be reduced by the protonation of the carbon atom with oxygen atom adsorbed on catalytic surface to produce *OCHO. This intermediate is then reduced to formate.^[^ ^98^ ^]^

**Figure 7:**
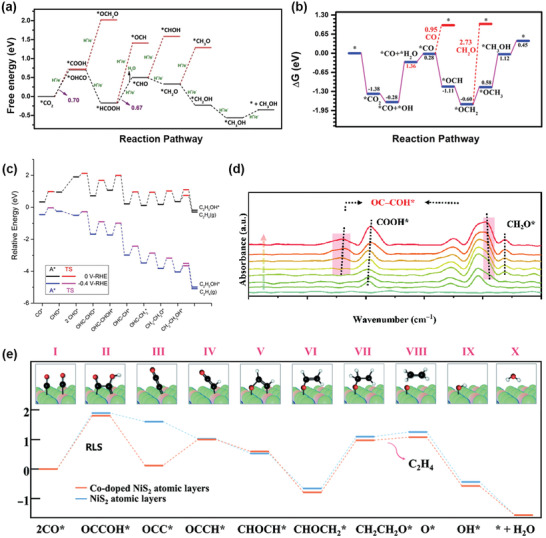
a) Free energy profiles for the CO_2_RR on Cu_3_(HHTQ)_2_. Reproduced with permission.^[^ ^100b^ ^]^ Copyright 2021, Wiley‐VCH. b) Free energy diagram of CO_2_RR on BP (111) surface. Reproduced with permission.^[^ ^101^ ^]^ Copyright 2019, Wiley‐VCH. c) Dominant path and associated reaction energetics identified for ethylene and ethanol production starting with CO* on the Cu(100) facet at 0 V versus RHE and at −0.4 V versus RHE. Energy values are referenced to the CO_2_(g), H^+^(aq) + e^−^ pair and a clean Cu(100) surface. Reproduced with permission.^[^ ^104^ ^]^ Copyright 2016, American Chemical Society. d) In situ FTIR spectra measurements of reaction intermediates over the Co‐doped NiS_2_ atomic layers. e) Free energy diagrams of CO_2_ reduction to C_2_H_4_ for the Co‐doped NiS_2_ atomic layers and NiS_2_ atomic layers. Reproduced with permission.^[^ ^108^ ^]^ Copyright 2021, Springer Nature.

Beyond the initial reduction products, the other C1/C2+ products can be derived from the further reduction of CO or HCOOH. HCOOH is generally accepted to be the key intermediate in the formation of formaldehyde.^[^ ^99^ ^]^ After the reduction of CO_2_ to formic acid, HCOOH is further reduced to formaldehyde via a sequential two‐electron transfer process. The reported pathways of the formation of methanol and methane are dissimilar. Some investigations have reported HCOOH as the key intermediate in the formation of CH_3_OH.^[^ ^100^ ^]^ For example, the DFT calculations (**Figure** **7**a) on 2D MOF Cu_3_(HHTQ)_2_ revealed that the hydrogenation of oxygen atoms to produce *HCOOH releases less energy than the hydrogenation of carbon atoms to form *OCH_2_O (0.88 vs 1.32 eV), indicating that the generation of the *HCOOH intermediate is thermodynamically favorable.^[^ ^100b^ ^]^ With one transferred electron and protonation at the oxygen atom, one molecule of H_2_O is released, and the *CHO intermediate is formed. Adsorbed *CH_2_O is considered another crucial CO_2_ reduction intermediate, and it is subsequently reduced to *CH_2_OH. Following further reduction with the transfer of one e^–^, *CH_3_OH is generated and then desorbs from the catalytic interface. Alternatively, a reaction pathway of CO_2_ reduction to CH_3_OH via the intermediate *CO has been reported on the nonmetal BP (111) catalytic surface.^[^ ^101^ ^]^ In contrast to the aforementioned mechanism, *OCH_2_ is regarded as another key intermediate in addition to *CO. DFT calculations (Figure 7b) indicated that the hydrogenation of *CO to *OCH is more favorable. With one transferred electron, *OCH is reduced to *OCH_2_ by protonation at the carbon atom, which results in a lower free energy change. The intermediate *OCH_2_ is then reduced to *OCH_3_ via one H^+^/e^–^ transfer to a carbon atom and is eventually reduced to *CH_3_OH via another single‐electron transfer step.

The formation of methane requires an eight‐electron transfer reaction, which is initiated by the reduction of the intermediate *CO to *CHO. There are two potential pathways for the formation of CH_4_ after the formation of *CHO. One is the *OCH_2_ pathway, which involves the formation of *OCH_2_ and *OCH_3_, in which the protonation of the carbon atom is favored and the O atom bonds with the catalytic surface. DFT calculations have shown that on a catalytic single‐atom Zn surface, the O atom bonds with the single Zn atom, and the C atom is protonated after the formation of *CHO, generating *OCH_2_ and *OCH_3_.^[^ ^102^ ^]^ Subsequently, *OCH_3_ is reduced to *OCH_4_, thus releasing a CH_4_ molecule and leaving *OH bonded to the catalyst surface. The final step is the adsorption of one more proton to *OH, producing H_2_O. The alternative pathway is the *CHOH pathway. Following the formation of *CHO, *CHOH forms via the preferred protonation of the O atom. For instance, on the iridium‐doped TiC catalyst, *CHOH is found to be more stable than *CH_2_O.^[^ ^103^ ^]^ With an electron transfer, *CHOH is reduced to *CH with the generation of one molecule of H_2_O. Then, CH_4_ forms through three sequential single‐electron transfers and the protonation of the carbon atom and then desorbs from the catalyst surface.

The formation of C2+ products is more complicated since it involves not only multielectron transfer but also C—C coupling. Although the pathways for the formation of C2+ products are elusive, it is generally accepted that CO_2_ is first reduced to adsorbed *CO, which is a key intermediate in the generation of C2+ products. Following the formation of *CO, C—C coupling, one of the most crucial steps in CO_2_ reduction to C2+ compounds, occurs. Three potential mechanistic pathways of C—C coupling are proposed. In the first pathway, C—C coupling occurs after the formation of the *CHO intermediate derived from the hydrogenation of carbon atoms by one‐electron transfer. *CHO preferentially forms on Cu(100), where C—C coupling to form C2 products is achieved by the dimerization of the *CHO species.^[^ ^104^ ^]^ The DFT calculations illustrated a barrier of 0.22 eV for the nonelectrochemical coupling of *CHO and *CHO, which is lower than that of the coupling of *CO and *CHO, indicating that the C—C coupling kinetics can be promoted by increasing the hydrogenation degree of C1 intermediates. Compared with the competitive conversion of *CHO to *CH_2_O which is the key intermediate for methanol and methane formation, adsorbed OHC—CHO is favored at low potentials since C—C coupling is a nonelectrochemical step. Following C—C coupling, a series of reduction reactions, including O—H bond formation, C—OH bond cleavage, and C—H bond formation, occur, producing C2 compounds (Figure 7c). The second pathway, the dimerization of *CO, is considered a more prevalent C—C coupling mechanism and is reported in most studies. Given that CO is one of the main products of CO_2_RR, surface coverage of *CO is supposed to be larger than that of hydrogenated species.^[^ ^105^ ^]^ Therefore, the C—C coupling via direct dimerization of *CO is more likely to occur due to the high surface concentration of *CO. This tendency can be verified by both electrochemical analysis and DFT calculations. For example, Meng et al.^[^ ^106^ ^]^ reported a tandem catalyst PTF(Ni)/Cu constructed by dispersing Cu NPs on the porphyrinic triazine framework anchored with atomically isolated Ni‐N_4_ sites for highly selective electrocatalytic CO_2_ to C_2_H_4_. Operando ATR‐FTIR studies confirmed the appearance of chemisorbed CO peak in the band located at 2089 cm^–1^. A large amount of *CO is expected to trigger the formation of C2+ products. Furthermore, a band at 1585 cm^–1^ was displayed, which can be assigned to the C—O stretching of *COCO^–^ intermediate. The bands were red shift in the ^13^CO_2_ labeling ATR‐FTIR experiments in KCl solution, indicating that the observed bands are ascribed to CO_2_RR intermediates instead of carbonate or bicarbonate from electrolytes. DFT calculations further revealed the tandem mechanism that CO can be desorbed from PTF(Ni) and then migrate to the adjacent Cu(200) surface due to the lower adsorption energy at Cu(200). With the surface coverage of CO increasing, CO dimerization for the formation of *OCCO with a significantly lower free energy (0.51 eV) is favored in comparison to the competitive hydrogenation of *CO for the generation of *CHO. After the rate‐determining step, C_2_H_4_ is produced via several intermediates such as *OCCOH, *CCO, and *CHCHO by a series of H^+^/e^–^ transfer reaction on Cu(200) surface. In addition to tandem catalysis, nanoconfinement also provides an effective strategy for the promotion of C—C coupling by tuning the diffusion kinetics to achieve a high local concentration of C1 intermediates for their dimerization. For instance, a series of Cu_2_O hollow multi‐shell structures with different shell numbers were prepared based on the finite‐element method simulation results, presenting a maximum C2+ FE of 77%. The nanoconfinement effect promotes the contact and interaction of reactants, meanwhile the restricted outflux of as‐formed species can suppress the desorption of C1 intermediates. Current‐step experiment and in situ electrochemical Raman spectroscopy studies unraveled that the increase of shell number leads to a higher coverage of surface‐absorbed CO for enhanced carbon dimerization.^[^ ^107^ ^]^ Another evidence supporting the pathway of direct *CO coupling is offered by mechanistic studies of CO_2_ photoreduction over Co‐doped NiS_2_ atomic layers (Figure 7d,e). Via in situ FTIR measurements, absorption bands were observed at approximately 1191 and 1672 cm^–1^ during photocatalytic CO_2_ reduction.^[^ ^108^ ^]^ These two peaks can be ascribed to the C—OH and C=O stretching modes of the OC—COH intermediate, which is formed through the dimerization of *CO and the protonation of the O atom. In the third pathway, C—C coupling may also occur between *CO and *CHO. Head‐Gorden and co‐workers proposed an alternative mechanism of C—C coupling between *CO and *CHO to derive C2 products over the (100) and (111) facets of Cu.^[^ ^109^ ^]^ This mechanism identifies *COCHO as the key intermediate in the reduction of CO_2_ to C2 products, as the DFT calculations indicate that at high potentials the reduction of CO to *CHO with subsequent coupling with *CO to form *COCHO is more favorable than the dimerization of *CO. In comparison to its tautomer *COCOH, the structure of *COCHO does not contain a double bond to the surface, thus presenting 0.43 eV more stable without an applied potential. These findings provide evidence for the proposed C—C bond formation mechanism.

### 3.2 Proton Coupling and Reaction Pathways

CO_2_ is thermodynamically stable and kinetically inert, making its reduction difficult.^[^ ^110^ ^]^ After the adsorption of CO_2_ molecules on the catalytic interface, the formation of CO_2_•^−^ anion radicals via a one‐electron transfer process requires a high overpotential of ‐1.90 V to convert the linear molecular structure of CO_2_ to a bent anionic radical.^[^ ^111^ ^]^ However, with proton coupling, the overpotential of the CO_2_RR shifts to a more positive value, indicating a lower energy barrier.^[^ ^112^ ^]^ This result suggests the significant role of protons in the reduction of CO_2_. In natural photosynthesis, protons are produced by the light reaction where water is oxidized and then transferred by the proton transferase NADPH, which can supply recyclable protons for the CO_2_RR. In the biomimetic PEC CO_2_ reduction, the source and supply route of protons can vary, and they include the dissociated protons provided by the electrolyte and the protons generated at the anode.

**Figure 8:**
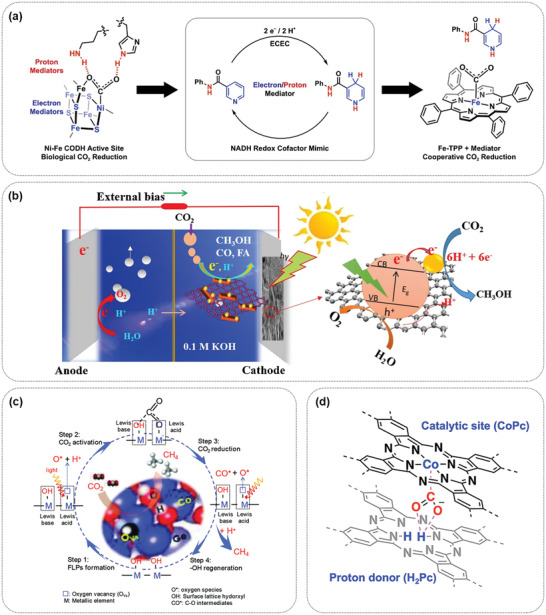
a) Bioinspired design of second‐sphere additives that enable dual electron and proton control for facilitating electrochemical CO_2_ reduction catalyzed by iron tetraphenylporphyrin (Fe‐TPP). Reproduced with permission.^[^ ^115^ ^]^ Copyright 2020, American Chemical Society. b) Proposed CO_2_ reduction mechanism over the Au/*α*‐Fe_2_O_3_/RGO photocathode. Reproduced with permission.^[^ ^117^ ^]^ Copyright 2021, Elsevier. c) Possible four‐step CH_4_ generation mechanism for using the surface lattice hydroxyl as solid‐state proton source. Reproduced with permission.^[^ ^118^ ^]^ Copyright 2018, Wiley‐VCH. d) Proposed proton‐donor mechanism of H_2_Pc for the synergistic catalysis of CO_2_ reduction. Reproduced with permission.^[^ ^119^ ^]^ Copyright 2021, Wiley‐VCH.

In aqueous electrolyte solution, water molecules are generally considered proton donors that participate in CO_2_ reduction. The mechanism by which water molecules near the cathode act as proton sources to promote the CO_2_RR is proposed at the MoS_2_ interface.^[^ ^113^ ^]^ DFT calculations imply that one proton dissociates from a water molecule near the cathode and then is transferred through the hydrogen bond chain; it then combines with *CO_2_ to form a *COOH intermediate with a relatively low free energy barrier of 0.21 eV. With the aid of the protons, the C—OH bond is broken to form *CO, simultaneously producing a H_2_O molecule. CO finally desorbs from the cathode surface, and the newly generated H_2_O molecule participates in the reaction again and further lowers the energy barrier. In addition to water, some organic molecules serve as proton donors to facilitate the CO_2_RR. For example, to suppress the competing HER, *n*‐propanol (PrOH) has been employed as a weak proton donor in CO_2_ reduction by FeTPP.^[^ ^114^ ^]^ The presence of the weak proton donor favors the protonation of the C atom of CO_2_, leading to the selective production of formate. Inspired by natural photosynthesis, researchers have investigated NADH analogs as redox mediators for the enhanced catalytic performance of electrochemical CO_2_ reduction by iron porphyrin (**Figure** **8**a).^[^ ^115^ ^]^ The redox reaction of NADH analogs can facilitate the two‐electron/two‐proton transfer process, supplying protons for the reduction of CO_2_ to CO via a cyclic pathway. With the addition of a proton/electron donor, the catalytic activity for CO_2_ reduction is enhanced 13‐fold. Further experiments with different additives have shown that employing an electron‐only additive enhances the rates of CO evolution over that of pristine Fe‐TPP (8.4‐fold), but these systems do not outperform NADH analogs that serve as both electron and proton sources, implying the significance of protons in improving the catalytic activity.

While they are often supplied by electrolytes or additives, protons can also be generated by water oxidation on the anode. For example, at a Nafion‐coated TiO_2_ interface, selective alcohol production was achieved due to the faster proton transport provided by the Nafion coating.^[^ ^116^ ^]^ In the PEC CO_2_ reduction system, protons are produced by the oxidization of water at the BiVO_4_ photoanode. At the cathode, abundant protons are monitored by the functional Nafion coating, which promotes the fast transfer of protons and high proton concentrations around the cathode. After the formation of the CO_2_•^−^ anion radical, a proton is transferred from the catholyte to CO_2_•^−^. Methanol and ethanol are generated through a series of proton‐assisted electron transfer processes. Since electron transfer is kinetically favorable over proton transport, the proton concentration around the cathode determines the reaction rates. Thus, the abundant protons accelerate the protonation of CO_2_•^−^ and the production of methanol by preventing the dimerization of CO_2_•^−^, whereas insufficient protons drive the dimerization of CO_2_•^−^ to generate more ethanol. This result indicates that protons play an important role in tuning the product selectivity of the CO_2_RR. To supply sufficient protons for CO_2_ reduction, water oxidation may be replaced by the oxidation of organics at the anode. For example, a paired PEC system reduces CO_2_ to CH_3_OH at the cathode and while also promoting the oxidation of furfural to 2‐furoic acid and 5‐hydroxyfuroic acid at the anode (Figure 8b).^[^ ^117^ ^]^ Under light irradiation, the photocathode is excited and produces photogenerated electrons and holes, while furfural, instead of water, is oxidized to generate more protons at the anode surface. These protons effectively participate in the PEC CO_2_ reduction to CH_3_OH, increasing the product yield.

To directly supply protons from the catholyte to the catalytic surface without undergoing proton transfer, catalysts can be endowed with proton donor functions by design. For instance, efficient CO_2_ reduction to CH_4_ with the aid of protons provided by the oxidation of lattice hydroxyls on CoGeO_2_(OH)_2_ photocatalyst surface has been reported (Figure 8c).^[^ ^118^ ^]^ Under light irradiation, the surface lattice hydroxyl groups were oxidized by the photogenerated holes to produce protons and oxygen vacancies. The adsorption and activation of CO_2_ were realized by capturing O atoms at the Lewis acid sites (the oxygen vacancies) and C atoms at the Lewis base sites (the hydroxyl groups). With the assistance of protons, the reaction kinetics of CO_2_RR were accelerated, promoting the formation of CH_4_. Instead of generating protons via the oxidization of surface hydroxyls, proton donor groups linked to the catalyst can also serve as proton sources. For example, the proton donor H_2_Pc can facilitate the selective CO_2_ reduction to CO on a conjugated composite microporous CoPc/H_2_Pc polymer catalyst (Figure 8d).^[^ ^119^ ^]^ The measurement of kinetic isotope effect (KIE) indicated that the rate‐determining step was the proton transfer process, and the addition of the proton donor, H_2_Pc, decreased the KIE value from 4.0 to 1.77, signifying accelerated proton transfer. The proton donor H_2_Pc lowered the energy barrier of the reduction of *CO_2_ to the *COOH intermediate and the further reduction of *COOH to *CO. Finally, CO was desorbed from the Co reactive sites. In another example, Barton Cole et al.^[^ ^120^ ^]^ investigated the homogeneous catalytic CO_2_ reduction by pyridinium. They found that pyridinium can reduce CO_2_ to CH_3_OH. Among these reaction pathways, the re‐adsorption of intermediates (•COOH, •CHO, CH_2_O) on pyridinium and the reaction of pyridinium radicals to provide H∙ were predominant. Theoretical calculations revealed that the Δ*G* of the adsorption of the intermediates on pyridinium was greater than 0, and the reaction between the intermediates and the pyridinium radical to generate a product was spontaneous. If hydrogen atoms were continuously added after CO_2_ adsorption, the ΔG of the adsorption process would remain less than 0.

Molecular catalysts can supply protons due to their specific molecular structures. The molecular catalyst system for CO_2_ reduction is also the type of catalysis that is most similar to natural photosynthesis. The direct addition of H atoms to C promotes the generation of value‐added C1 compounds. If the molecular catalyst can not only effectively adsorb CO_2_ but also provide the H^–^ that combines with C atoms, C1 compounds such as methane and methanol may be generated effectively. The biological coenzyme NADH, which is essential for the citric acid cycle, can provide one H^+^ and two e^–^ (equivalent to one H^–^). Tanaka's research team found that [Ru(pbn)(bpy)_2_](PF_6_)_2_ (pbn = 2‐(2‐pyridyl)benzo[b]‐1.5‐naphthyridine, bpy = 2,2’‐bipyridine) can be reduced to [Ru(pbnHH)(bpy)_2_](PF_6_)_2_; the structure of this complex is analogous to the coenzyme NADH under electrochemical conditions. It was also found that the Ru complex can transfer hydrides between molecules; it can directly transfer H^–^ to the C atom in the carbonyl group of acetone. Subsequently, the research team studied the catalytic ability of [Ru(pbnHH)(bpy)_2_]^2+^ in CO_2_ reduction.^[^ ^121^ ^]^ The C—H bonds can be formed through the transfer of H^–^ with the aid of the benzoic acid anion, thereby reducing CO_2_ to formic acid. Moreover, [Ru(pbn)(bpy)_2_]^2+^ can be reduced again to [Ru(pbnHH)(bpy)_2_]^2+^ by photocatalytic reduction; this is an example of molecular catalyst recycling. However, this type of molecular catalyst cannot provide abundant CO_2_ adsorption sites and can only achieve the intermolecular transfer of H^–^. If intramolecular H^–^ transfer can be achieved, the catalytic activity of the molecular catalyst may be further enhanced.

To compare the hydride transfer capacity of different molecular catalysts, Muckerman et al.^[^ ^122^ ^]^ then calculated the hydricity of [Ru(pbnHH)(bpy)_2_]^2+^ and other molecular catalysts. The calculated results showed that the hydride‐donating ability of [Ru(pbnHH)(bpy)_2_]^2+^ is not very strong, while the triply reduced and doubly protonated isomeric [Ru(pbnHH)(bpy)_2_^•–^]^2+^ exhibited the most promising hydride‐donating power. Moreover, it was found that the hydride transfer capacity of [Re(pbnHH)(CO)_3_Cl] is stronger than that of [Ru(pbnHH)(bpy)_2_]^2+^. According to the previous review on the Re bipyridine complex, during the catalytic reduction process, this type of molecular catalysts can easily lose a Cl^–^ to form a five‐coordinate compound, which provides an empty coordination site for the combination of CO_2_ with the complex.^[^ ^123^ ^]^ In this way, the M—C bond can be formed and while intramolecular H^–^ transfer occurs simultaneously.

In summary, during CO_2_ reduction, as protons are coupled, the energy barrier of CO_2_RR can be lowered and the catalytic reaction pathway can be altered, facilitating the conversion of CO_2_ with low energy consumption and high efficiency. The protons can not only be derived from the electrolyte and anodic oxidation reaction but also be provided directly by the catalyst. Additives may be added as extra proton donors, or the anodic water oxidation reaction can be replaced by the oxidization of organic compounds. However, the proton transfer efficiency is still limited by the diffusion rate more so than when protons are directly provided by catalyst. Therefore, to construct an excellent biomimetic PEC interface, it is necessary to consider the integration of enhanced CO_2_ adsorption, multisite activation, electron transfer, and proton coupling functions.

## 4 Conclusions and Perspectives

Converting the greenhouse gas CO_2_ to value‐added fuels and chemicals is a promising way to alleviate two global issues, energy shortages, and environmental deterioration. The efficient CO_2_ reduction reaction relies on the construction of catalytic interfaces and explorations of the underlying reaction mechanisms. Natural photosynthesis provides inspiration for the construction of catalytic interfaces to promote CO_2_ adsorption, activation, and CO_2_ conversion. Mimicking natural photosynthesis, biomimetic PEC exhibits the advantages of EC, which include oriented electron transfer, and the strengths of PC, which include reduced electrical energy consumption; therefore, this process has great potential for CO_2_ utilization. This review illustrates strategies for constructing biomimetic interfaces with enhanced CO_2_ adsorption, activation, and conversion. It describes catalyst modification, biomimetic catalytic interface design, electron transfer on the catalytic interface, and reaction mechanisms involving proton coupling and electron transfer to form different products. It provides a reference for the design and optimization of efficient biomimetic PEC systems. Among five biomimetic PEC interfaces, metal cocatalysts can broaden the photoresponse range of semiconductors and promote the rapid separation of photogenerated carriers. Furthermore, the controllable nanoparticle morphology and size and adjustable crystal facets enable the efficient activation of CO_2_. Nevertheless, the adsorption of CO_2_ at the metal surface is insufficient compared to the other four types of PEC interfaces. The modification of small molecules can introduce acid‐base active sites via functional groups such as —OH and —NH—, thereby enhancing the adsorption and activation of CO_2_. By modifying the central metal atom and regulating the ligands, molecular catalysts exhibit high controllability of catalytic properties, but the products are often limited to C1 compounds such as CO and HCOOH. The MOF/semiconductor biomimetic PEC interface integrates the advantages of the above three types of hybrid catalytic interfaces. It is designable in terms of light absorption and CO_2_ adsorption and activation, presenting great potential in realizing a thorough simulation of natural photosynthesis. The microorganism/semiconductor biomimetic interface enables the production of long‐chain multicarbon compounds via the assistance of microbial metabolism. However, the product selectivity and reduction efficiency are limited by the selection of microorganisms and the harsh cultivation environments. Although many investigations on biomimetic PEC CO_2_ reduction and catalytic mechanisms have made great breakthroughs, challenges still remain and substantial efforts are required to further enhance the conversion rate and selectivity of CO_2_RR.

First, the linkage between the decoration material and the semiconductor is usually a noncovalent interaction that is susceptible to leaching and losing its activity. Covalent attachment is a promising way to overcome this issue, which requires a stronger link between the catalysts and semiconductor substrates. Several strategies for fabricating hybrid PEC interfaces by covalent linkage assembly include chemical coordination, introducing anchoring groups (such as carboxylic acids, phosphonic acids, and hydroxamic acids) and electrochemical polymerization. Chemical bonding ensures efficient charge transfer between semiconductor electrodes and catalysts, thus facilitating the rapid conversion of CO_2_.

Second, the reduction of CO_2_ to C1 compounds such as CO and formate can be achieved with a high selectivity on the reported PEC interfaces, though the selectivity of C2 products is still limited by the complex reaction pathways involving not only proton coupling and electron transfer processes but also C—C coupling. Although the formation of multicarbon compounds is challenging, natural photosynthesis can realize the oriented conversion of CO_2_ to glucose, which indicates plenty of scope for investigations on mimicking natural photosynthesis to obtain multicarbon products. In addition to mimicking photosynthesis to enhance CO_2_ fixation and activation on the PEC interface, emerging catalytic strategies such as tandem catalysis, confinement engineering, highly dispersed single‐ or dual‐atom catalysts can be adopted to increase the local concentration of C1 intermediates, thus making the formation of C—C bond more likely to occur.

Third, since CO_2_ reduction on the biomimetic PEC interface is a complex process including both the multielectron transfer from the catalytic surface to the adsorbed CO_2_ molecules and the interfacial electron transfer between the catalyst and semiconductor, determining the underlying mechanism is challenging, especially for the formation of C2+ compounds; a lack of mechanistic understanding impedes the rational design of effective PEC interfaces. Although plenty of experimental efforts have been dedicated to identifying the potential reaction intermediates and pathways toward C2+ products, currently only a few species have been detected. Due to their high reactivity, the surface coverage of these intermediates can be extremely low at all applied potentials, making their detection challenging. Advanced characterization techniques, including operando surface‐enhanced Raman scattering, attenuated total reflection (ATR)‐IR, scanning electrochemical microscopy (SECM), and in situ X‐ray absorption spectroscopy, should be employed to provide key evidence for the proposed reaction mechanisms. These experimental methods combined with theoretical calculations enable the determination of the reaction intermediates, reactive sites and the potential reaction pathways.

Although PC can reduce CO_2_ without additional energy input, directed electron transfer does not occur. There is still a long way to go to fully utilize PC to simulate natural photosynthesis. EC can achieve directed electron transfer and supply electrons in a cycle, but much electrical energy is required owing to the high overpotential of CO_2_RR. Complementing the advantages of PC and EC, we consider that PEC CO_2_ reduction is one of the most promising ways of thoroughly simulating natural photosynthesis in the short term. We believe that this review can provide researchers with inspiration for designing efficient biomimetic PEC interfaces to achieve a higher yield and better selectivity of CO_2_ reduction products.

## Conflict of Interest

The authors declare no conflict of interest.

## References

[1] A. Otto , T. Grube , S. Schiebahn , D. Stolten , Energy Environ. Sci. 2015, 8, 3283.

[2] W. Lu , Y. Zhang , J. Zhang , P. Xu , Ind. Eng. Chem. Res. 2020, 59, 5536.

[3] R. Shi , G. I. Waterhouse , T. Zhang , Sol. RRL 2017, 1, 1700126.

[4] a) L. Zhang , Z.‐J. Zhao , J. Gong , Angew. Chem., Int. Ed. 2017, 56, 11326; 10.1002/anie.201612214 28168799

[5] P. Prabhu , V. Jose , J.‐M. Lee , Adv. Funct. Mater. 2020, 30, 1910768.

[6] A. Kumar , V. Hasija , A. Sudhaik , P. Raizada , Q. Van Le , P. Singh , T.‐H. Pham , T. Kim , S. Ghotekar , V.‐H. Nguyen , Chem. Eng. J. 2022, 430, 133031.

[7] a) B. Zhu , K. Qiu , C. Shang , Z. Guo , J. Mater. Chem. A 2015, 3, 5212;

[8] a) X. Liu , S. Inagaki , J. Gong , Angew. Chem., Int. Ed. 2016, 55, 14924; 10.1002/anie.201600395 27739176

[9] A. M. Appel , J. E. Bercaw , A. B. Bocarsly , H. Dobbek , D. L. DuBois , M. Dupuis , J. G. Ferry , E. Fujita , R. Hille , P. J. Kenis , C. A. Kerfeld , R. H. Morris , C. H. Peden , A. R. Portis , S. W. Ragsdale , T. B. Rauchfuss , J. N. Reek , L. C. Seefeldt , R. K. Thauer , G. L. Waldrop , Chem. Rev. 2013, 113, 6621. 23767781 10.1021/cr300463y PMC3895110

[10] a) Y. X. Pan , Y. You , S. Xin , Y. Li , G. Fu , Z. Cui , Y. L. Men , F. F. Cao , S. H. Yu , J. B. Goodenough , J. Am. Chem. Soc. 2017, 139, 4123; 28215081 10.1021/jacs.7b00266

[11] a) R. R. Ikreedeegh , M. Tahir , J. CO2 Util. 2021, 43, 101381;

[12] X. Yang , D. Wang , ACS Appl. Energy Mater. 2018, 1, 6657.

[13] K. Wang , Y. Ma , Y. Liu , W. Qiu , Q. Wang , X. Yang , M. Liu , X. Qiu , W. Li , J. Li , Green Chem. 2021, 23, 3207.

[14] G. Hyun , J. T. Song , C. Ahn , Y. Ham , D. Cho , J. Oh , S. Jeon , Proc. Natl. Acad. Sci. USA 2020, 117, 5680. 32132207 10.1073/pnas.1918837117 PMC7084147

[15] G. Collins , E. Armstrong , D. McNulty , S. O'Hanlon , H. Geaney , C. O'Dwyer , Sci. Technol. Adv. Mater. 2016, 17, 563. 27877904 10.1080/14686996.2016.1226121 PMC5111560

[16] A. M. Appel , J. E. Bercaw , A. B. Bocarsly , H. Dobbek , D. L. DuBois , M. Dupuis , J. G. Ferry , E. Fujita , R. Hille , P. J. Kenis , Chem. Rev. 2013, 113, 6621. 23767781 10.1021/cr300463y PMC3895110

[17] a) X. Li , J. Yu , M. Jaroniec , X. Chen , Chem. Rev. 2019, 119, 3962; 30763077 10.1021/acs.chemrev.8b00400

[18] a) V. Kumaravel , J. Bartlett , S. C. Pillai , ACS Energy Lett. 2020, 5, 486;

[19] a) G. Wang , J. Chen , Y. Ding , P. Cai , L. Yi , Y. Li , C. Tu , Y. Hou , Z. Wen , L. Dai , Chem. Soc. Rev. 2021, 50, 4993;33625419

[20] a) J. Ferreira de Brito , P. G. Corradini , A. B. Silva , L. H. Mascaro , ChemElectroChem 2021, 8, 4305;

[21] W. Zhang , Z. Jin , Z. Chen , Adv. Sci. 2022, 9, 2105204. 10.1002/advs.202105204 PMC8948570 35072349

[22] D. Ješić , D. L. Jurković , A. Pohar , L. Suhadolnik , B. Likozar , Chem. Eng. J. 2021, 407, 126799.

[23] X. Chang , T. Wang , P. Yang , G. Zhang , J. Gong , Adv. Mater. 2019, 31, 1804710. 10.1002/adma.201804710 30537099

[24] J. Ran , M. Jaroniec , S. Z. Qiao , Adv. Mater. 2018, 30, 1704649. 10.1002/adma.201704649 29315885

[25] I. Shown , H.‐C. Hsu , Y.‐C. Chang , C.‐H. Lin , P. K. Roy , A. Ganguly , C.‐H. Wang , J.‐K. Chang , C.‐I. Wu , L.‐C. Chen , Nano Lett. 2014, 14, 6097. 25354234 10.1021/nl503609v

[26] W. Hou , W. H. Hung , P. Pavaskar , A. Goeppert , M. Aykol , S. B. Cronin , ACS Catal. 2011, 1, 929.

[27] Y. Wang , J. Yu , W. Xiao , Q. Li , J. Mater. Chem. A 2014, 2, 3847.

[28] R. Lee , Y. Kumaresan , S. Y. Yoon , S. H. Um , I. K. Kwon , G. Y. Jung , RSC Adv. 2017, 7, 7469.

[29] W.‐N. Wang , W.‐J. An , B. Ramalingam , S. Mukherjee , D. M. Niedzwiedzki , S. Gangopadhyay , P. Biswas , J. Am. Chem. Soc. 2012, 134, 11276. 22694165 10.1021/ja304075b

[30] S. Bai , X. Wang , C. Hu , M. Xie , J. Jiang , Y. Xiong , Chem. Commun. 2014, 50, 6094. 10.1039/c4cc00745j 24777281

[31] Q. Lu , F. Jiao , Nano Energy 2016, 29, 439.

[32] a) K. P. Kuhl , E. R. Cave , D. N. Abram , T. F. Jaramillo , Energy Environ. Sci. 2012, 5, 7050;

[33] F. Studt , I. Sharafutdinov , F. Abild‐Pedersen , C. F. Elkjær , J. S. Hummelshøj , S. Dahl , I. Chorkendorff , J. K. Nørskov , Nat. Chem. 2014, 6, 320. 24651199 10.1038/nchem.1873

[34] P. Hirunsit , J. Phys. Chem. C 2013, 117, 8262.

[35] D. Kim , J. Resasco , Y. Yu , A. M. Asiri , P. Yang , Nat. Commun. 2014, 5, 4948. 25208828 10.1038/ncomms5948

[36] S. Ma , M. Sadakiyo , M. Heima , R. Luo , R. T. Haasch , J. I. Gold , M. Yamauchi , P. J. Kenis , J. Am. Chem. Soc. 2017, 139, 47. 27958727 10.1021/jacs.6b10740

[37] X. Sun , Q. Zhu , X. Kang , H. Liu , Q. Qian , Z. Zhang , B. Han , Angew. Chem., Int. Ed. 2016, 55, 6771. 10.1002/anie.201603034 27098284

[38] S. t. Neaţu , J. A. Maciá‐Agulló , P. Concepción , H. Garcia , J. Am. Chem. Soc. 2014, 136, 15969. 25329687 10.1021/ja506433k

[39] Q. Shen , J. Ma , X. Huang , N. Yang , G. Zhao , Appl. Catal., B 2017, 219, 45.

[40] Q. Kang , T. Wang , P. Li , L. Liu , K. Chang , M. Li , J. Ye , Angew. Chem., Int. Ed. 2015, 54, 841. 10.1002/anie.201409183 25422137

[41] Q. Chen , X. Chen , M. Fang , J. Chen , Y. Li , Z. Xie , Q. Kuang , L. Zheng , J. Mater. Chem. A 2019, 7, 1334.

[42] Z. Sun , W. Fang , L. Zhao , H. Wang , Appl. Surf. Sci. 2020, 504, 144347.

[43] L. J. Murphy , K. N. Robertson , R. A. Kemp , H. M. Tuononen , J. A. Clyburne , Chem. Commun. 2015, 51, 3942. 10.1039/c4cc08510h 25601453

[44] H. Yu , R. Shi , Y. Zhao , G. I. Waterhouse , L. Z. Wu , C. H. Tung , T. Zhang , Adv. Mater. 2016, 28, 9454. 27623955 10.1002/adma.201602581

[45] S. Guo , S. Zhao , J. Gao , C. Zhu , X. Wu , Y. Fu , H. Huang , Y. Liu , Z. Kang , Nanoscale 2017, 9, 298. 27910981 10.1039/c6nr08104e

[46] M. S. Xie , B. Y. Xia , Y. Li , Y. Yan , Y. Yang , Q. Sun , S. H. Chan , A. Fisher , X. Wang , Energy Environ. Sci. 2016, 9, 1687.

[47] P. Xia , B. Zhu , J. Yu , S. Cao , M. Jaroniec , J. Mater. Chem. A 2017, 5, 3230.

[48] Q. Zhu , Y. Cao , Y. Tao , T. Li , Y. Zhang , H. Shang , J. Song , G. Li , J. CO2 Util. 2021, 54, 101781.

[49] C. Kim , T. Eom , M. S. Jee , H. Jung , H. Kim , B. K. Min , Y. J. Hwang , ACS Catal. 2017, 7, 779.

[50] Z. Sun , J. M. T. A. Fischer , Q. Li , J. Hu , Q. Tang , H. Wang , Z. Wu , M. Hankel , D. J. Searles , L. Wang , Appl. Catal., B 2017, 216, 146.

[51] D. Yang , H. Yu , T. He , S. Zuo , X. Liu , H. Yang , B. Ni , H. Li , L. Gu , D. Wang , Nat. Commun. 2019, 10, 3844. 31451689 10.1038/s41467-019-11817-2 PMC6710284

[52] a) B. J. Fisher , R. Eisenberg , J. Am. Chem. Soc. 1980, 102, 7361;

[53] a) J. Hawecker , J.‐M. Lehn , R. Ziessel , J. Chem. Soc., Chem. Commun. 1983, 536;

[54] a) D. L. DuBois , A. Miedaner , R. C. Haltiwanger , J. Am. Chem. Soc. 1991, 113, 8753;

[55] a) J.‐H. Jeoung , H. Dobbek , Science 2007, 318, 1461; 18048691 10.1126/science.1148481

[56] S. Sato , T. Morikawa , S. Saeki , T. Kajino , T. Motohiro , Angew. Chem., Int. Ed. 2010, 49, 5101. 10.1002/anie.201000613 20607873

[57] X. Huang , Q. Shen , J. Liu , N. Yang , G. Zhao , Energy Environ. Sci. 2016, 9, 3161.

[58] T. M. Suzuki , H. Tanaka , T. Morikawa , M. Iwaki , S. Sato , S. Saeki , M. Inoue , T. Kajino , T. Motohiro , Chem. Commun. 2011, 47, 8673. 10.1039/c1cc12491a 21713249

[59] S. A. Yao , R. E. Ruther , L. Zhang , R. A. Franking , R. J. Hamers , J. F. Berry , J. Am. Chem. Soc. 2012, 134, 15632. 22963046 10.1021/ja304783j

[60] C. L. Anfuso , R. C. Snoeberger III , A. M. Ricks , W. Liu , D. Xiao , V. S. Batista , T. Lian , J. Am. Chem. Soc. 2011, 133, 6922. 21504161 10.1021/ja2013664

[61] K. Sekizawa , K. Maeda , K. Domen , K. Koike , O. Ishitani , J. Am. Chem. Soc. 2013, 135, 4596. 23470246 10.1021/ja311541a PMC3679556

[62] S. Oh , J. R. Gallagher , J. T. Miller , Y. Surendranath , J. Am. Chem. Soc. 2016, 138, 1820. 26804469 10.1021/jacs.5b13080

[63] M. Schreier , J. Luo , P. Gao , T. Moehl , M. T. Mayer , M. Grätzel , J. Am. Chem. Soc. 2016, 138, 1938. 26804626 10.1021/jacs.5b12157

[64] J. Liu , H. Shi , Q. Shen , C. Guo , G. Zhao , Green Chem. 2017, 19, 5900.

[65] a) A. Kumar , D. G. Madden , M. Lusi , K. J. Chen , E. A. Daniels , T. Curtin , J. J. Perry IV , M. J. Zaworotko , Angew. Chem., Int. Ed. 2015, 54, 14372; 10.1002/anie.201506952 26440308

[66] M. Fernandez , P. G. Boyd , T. D. Daff , M. Z. Aghaji , T. K. Woo , J. Phys. Chem. Lett. 2014, 5, 3056. 26278259 10.1021/jz501331m

[67] J. Albo , D. Vallejo , G. Beobide , O. Castillo , P. Castaño , A. Irabien , ChemSusChem 2017, 10, 1100. 27557788 10.1002/cssc.201600693

[68] R. Shimoni , Z. Shi , S. Binyamin , Y. Yang , I. Liberman , R. Ifraemov , S. Mukhopadhyay , L. Zhang , I. Hod , Angew. Chem., Int. Ed. 2022, 61, e202206085. 10.1002/anie.202206085 PMC9401588 35674328

[69] J.‐D. Yi , D.‐H. Si , R. Xie , Q. Yin , M.‐D. Zhang , Q. Wu , G.‐L. Chai , Y.‐B. Huang , R. Cao , Angew. Chem., Int. Ed. 2021, 60, 17108. 10.1002/anie.202104564 34033203

[70] Y. Fu , D. Sun , Y. Chen , R. Huang , Z. Ding , X. Fu , Z. Li , Angew. Chem., Int. Ed. 2012, 51, 3364. 10.1002/anie.201108357 22359408

[71] Q. Liu , Z.‐X. Low , L. Li , A. Razmjou , K. Wang , J. Yao , H. Wang , J. Mater. Chem. A 2013, 1, 11563.

[72] R. Li , J. Hu , M. Deng , H. Wang , X. Wang , Y. Hu , H. L. Jiang , J. Jiang , Q. Zhang , Y. Xie , Adv. Mater. 2014, 26, 4783. 24861956 10.1002/adma.201400428

[73] S. Wang , J. Lin , X. Wang , Phys. Chem. Chem. Phys. 2014, 16, 14656. 24921181 10.1039/c4cp02173h

[74] S. Wang , X. Wang , Appl. Catal., B 2015, 162, 494.

[75] D. Tan , J. Zhang , L. Yao , X. Tan , X. Cheng , Q. Wan , B. Han , L. Zheng , J. Zhang , Nano Res. 2020, 13, 768.

[76] a) H. Jung , S. Y. Lee , C. W. Lee , M. K. Cho , D. H. Won , C. Kim , H.‐S. Oh , B. K. Min , Y. J. Hwang , J. Am. Chem. Soc. 2019, 141, 4624; 30702874 10.1021/jacs.8b11237

[77] F. Bayat , S. Sheibani , Mater. Res. Bull. 2022, 145, 111561.

[78] R. Gusain , P. Kumar , O. P. Sharma , S. L. Jain , O. P. Khatri , Appl. Catal., B 2016, 181, 352.

[79] Y. A. Wu , I. McNulty , C. Liu , K. C. Lau , Q. Liu , A. P. Paulikas , C.‐J. Sun , Z. Cai , J. R. Guest , Y. Ren , V. Stamenkovic , L. A. Curtiss , Y. Liu , T. Rajh , Nat. Energy 2019, 4, 957.

[80] H. Wu , X. Y. Kong , X. Wen , S. P. Chai , E. C. Lovell , J. Tang , Y. H. Ng , Angew. Chem., Int. Ed. 2021, 60, 8455. 10.1002/anie.202015735 33368920

[81] R. K. Yadav , G. H. Oh , N.‐J. Park , A. Kumar , K.‐j. Kong , J.‐O. Baeg , J. Am. Chem. Soc. 2014, 136, 16728. 25405924 10.1021/ja509650r

[82] N. Kornienko , J. Z. Zhang , K. K. Sakimoto , P. Yang , E. Reisner , Nat. Nanotechnol. 2018, 13, 890. 30291349 10.1038/s41565-018-0251-7

[83] a) H.‐Y. Yang , B.‐L. Bao , J. Liu , Y. Qin , Y.‐R. Wang , K.‐Z. Su , J.‐C. Han , Y. Mu , Bioelectrochemistry 2018, 119, 180; 29054074 10.1016/j.bioelechem.2017.10.002

[84] C. Liu , J. J. Gallagher , K. K. Sakimoto , E. M. Nichols , C. J. Chang , M. C. Chang , P. Yang , Nano Lett. 2015, 15, 3634. 25848808 10.1021/acs.nanolett.5b01254 PMC5812269

[85] K. K. Sakimoto , A. B. Wong , P. Yang , Science 2016, 351, 74. 26721997 10.1126/science.aad3317

[86] E. M. Nichols , J. J. Gallagher , C. Liu , Y. Su , J. Resasco , Y. Yu , Y. Sun , P. Yang , M. C. Chang , C. J. Chang , Proc. Natl. Acad. Sci. USA 2015, 112, 11461. 26305947 10.1073/pnas.1508075112 PMC4577177

[87] C. Liu , B. C. Colón , M. Ziesack , P. A. Silver , D. G. Nocera , Science 2016, 352, 1210. 27257255 10.1126/science.aaf5039

[88] G. Liu , F. Zheng , J. Li , G. Zeng , Y. Ye , D. M. Larson , J. Yano , E. J. Crumlin , J. W. Ager , L.‐w. Wang , F. M. Toma , Nat. Energy 2021, 6, 1124.

[89] Y. Zhang , D. Pan , Y. Tao , H. Shang , D. Zhang , G. Li , H. Li , Adv. Funct. Mater. 2022, 32, 2109600.

[90] Q. Shen , X. Huang , J. Liu , C. Guo , G. Zhao , Appl. Catal., B 2017, 201, 70.

[91] C. Jiang , S. J. A. Moniz , A. Wang , T. Zhang , J. Tang , Chem. Soc. Rev. 2017, 46, 4645. 28644493 10.1039/c6cs00306k

[92] a) Y. Zhu , Z. Xu , Q. Lang , W. Jiang , Q. Yin , S. Zhong , S. Bai , Appl. Catal., B 2017, 206, 282;

[93] a) K. M. Cho , K. H. Kim , K. Park , C. Kim , S. Kim , A. Al‐Saggaf , I. Gereige , H.‐T. Jung , ACS Catal. 2017, 7, 7064;

[94] a) K. Maeda , Adv. Mater. 2019, 31, 1808205; 10.1002/adma.201808205 31066136

[95] a) W. Zhu , C. Zhang , Q. Li , L. Xiong , R. Chen , X. Wan , Z. Wang , W. Chen , Z. Deng , Y. Peng , Appl. Catal., B 2018, 238, 339;

[96] a) H. Zhang , H. Liu , Z. Tian , D. Lu , Y. Yu , S. Cestellos‐Blanco , K. K. Sakimoto , P. Yang , Nat. Nanotechnol. 2018, 13, 900; 30275495 10.1038/s41565-018-0267-z

[97] J. Shen , R. Kortlever , R. Kas , Y. Y. Birdja , O. Diaz‐Morales , Y. Kwon , I. Ledezma‐Yanez , K. J. P. Schouten , G. Mul , M. T. Koper , Nat. Commun. 2015, 6, 8177. 26324108 10.1038/ncomms9177 PMC4569799

[98] Q. Gong , P. Ding , M. Xu , X. Zhu , M. Wang , J. Deng , Q. Ma , N. Han , Y. Zhu , J. Lu , Nat. Commun. 2019, 10, 2807. 31243275 10.1038/s41467-019-10819-4 PMC6594929

[99] a) K. Nakata , T. Ozaki , C. Terashima , A. Fujishima , Y. Einaga , Angew. Chem., Int. Ed. 2014, 53, 871; 10.1002/anie.201308657 24281847

[100] a) S. Xu , L. Li , E. A. Carter , J. Am. Chem. Soc. 2018, 140, 16749; 30398873 10.1021/jacs.8b09946

[101] S. Mou , T. Wu , J. Xie , Y. Zhang , L. Ji , H. Huang , T. Wang , Y. Luo , X. Xiong , B. Tang , Adv. Mater. 2019, 31, 1903499. 10.1002/adma.201903499 31338908

[102] L. Han , S. Song , M. Liu , S. Yao , Z. Liang , H. Cheng , Z. Ren , W. Liu , R. Lin , G. Qi , J. Am. Chem. Soc. 2020, 142, 12563. 32536159 10.1021/jacs.9b12111

[103] S. Back , Y. Jung , ACS Energy Lett. 2017, 2, 969.

[104] W. Luo , X. Nie , M. J. Janik , A. Asthagiri , ACS Catal. 2016, 6, 219.

[105] T. K. Todorova , M. W. Schreiber , M. Fontecave , ACS Catal. 2019, 10, 1754.

[106] D.‐L. Meng , M.‐D. Zhang , D.‐H. Si , M.‐J. Mao , Y. Hou , Y.‐B. Huang , R. Cao , Angew. Chem., Int. Ed. 2021, 60, 25485. 10.1002/anie.202111136 34533874

[107] C. Liu , M. Zhang , J. Li , W. Xue , T. Zheng , C. Xia , J. Zeng , Angew. Chem., Int. Ed. 2022, 61, e202113498. 10.1002/anie.202113498 34821457

[108] W. Shao , X. Li , J. Zhu , X. Zu , L. Liang , J. Hu , Y. Pan , J. Zhu , W. Yan , Y. Sun , Nano Res. 2022, 15, 1882.

[109] A. J. Garza , A. T. Bell , M. Head‐Gordon , ACS Catal. 2018, 8, 1490.

[110] W. Gao , S. Liang , R. Wang , Q. Jiang , Y. Zhang , Q. Zheng , B. Xie , C. Y. Toe , X. Zhu , J. Wang , L. Huang , Y. Gao , Z. Wang , C. Jo , Q. Wang , L. Wang , Y. Liu , B. Louis , J. Scott , A.‐C. Roger , R. Amal , H. He , S.‐E. Park , Chem. Soc. Rev. 2020, 49, 8584. 33073812 10.1039/d0cs00025f

[111] J. Wu , Y. Huang , W. Ye , Y. Li , Adv. Sci. 2017, 4, 1700194. 10.1002/advs.201700194 PMC5700640 29201614

[112] X. Cao , D. Tan , B. Wulan , K. Hui , K. Hui , J. Zhang , Small Methods 2021, 5, 2100700. 10.1002/smtd.202100700 34927933

[113] M. Asadi , M. H. Motevaselian , A. Moradzadeh , L. Majidi , M. Esmaeilirad , T. V. Sun , C. Liu , R. Bose , P. Abbasi , P. Zapol , Adv. Energy Mater. 2019, 9, 1803536.

[114] C. G. Margarit , N. G. Asimow , C. Costentin , D. G. Nocera , ACS Energy Lett. 2019, 5, 72.

[115] P. T. Smith , S. Weng , C. J. Chang , Inorg. Chem. 2020, 59, 9270. 32623894 10.1021/acs.inorgchem.0c01162

[116] M. J. Kang , C. W. Kim , A. U. Pawar , H. G. Cha , S. Ji , W.‐B. Cai , Y. S. Kang , ACS Energy Lett. 2019, 4, 1549.

[117] G. Bharath , K. Rambabu , A. Hai , N. Ponpandian , J. E. Schmidt , D. D. Dionysiou , M. A. Haija , F. Banat , Appl. Catal., B 2021, 298, 120520.

[118] X. Wang , L. Lu , B. Wang , Z. Xu , Z. Xin , S. Yan , Z. Geng , Z. Zou , Adv. Funct. Mater. 2018, 28, 1804191.

[119] R. Wang , X. Wang , W. Weng , Y. Yao , P. Kidkhunthod , C. Wang , Y. Hou , J. Guo , Angew. Chem., Int. Ed. 2022, 61, e202115503. 10.1002/anie.202115503 34851556

[120] E. Barton Cole , P. S. Lakkaraju , D. M. Rampulla , A. J. Morris , E. Abelev , A. B. Bocarsly , J. Am. Chem. Soc. 2010, 132, 11539. 20666494 10.1021/ja1023496

[121] H. Ohtsu , K. Tanaka , Angew. Chem., Int. Ed. 2012, 51, 9792. 10.1002/anie.201204348 22945431

[122] J. T. Muckerman , P. Achord , C. Creutz , D. E. Polyansky , E. Fujita , Proc. Natl. Acad. Sci. USA 2012, 109, 15657. 22826261 10.1073/pnas.1201026109 PMC3465420

[123] A. J. Morris , G. J. Meyer , E. Fujita , Acc. Chem. Res. 2009, 42, 1983. 19928829 10.1021/ar9001679

